# Paternal exposure to a common pharmaceutical (Ritalin) has transgenerational effects on the behaviour of Trinidadian guppies

**DOI:** 10.1038/s41598-021-83448-x

**Published:** 2021-02-17

**Authors:** Alex R. De Serrano, Kimberly A. Hughes, F. Helen Rodd

**Affiliations:** 1 Department of Ecology and Evolutionary Biology, University of Toronto, 25 Willcocks St, Toronto, ON M5S 3B2 Canada; 2 Department of Biological Science, Florida State University, 319 Stadium Dr, Tallahassee, FL 32304 USA

**Keywords:** Epigenetics, Behavioural ecology, Evolutionary ecology, Animal behaviour, Developmental disorders

## Abstract

Evidence is emerging that paternal effects, the nongenetic influence of fathers on their offspring, can be transgenerational, spanning several generations. Methylphenidate hydrochloride (MPH; e.g. Ritalin) is a dopaminergic drug that is highly prescribed to adolescent males for the treatment of Attention-deficit/hyperactivity disorder. It has been suggested that MPH could cause transgenerational effects because MPH can affect the male germline in rodents and because paternal effects have been observed in individuals taking similar drugs (e.g. cocaine). Despite these concerns, the transgenerational effects of paternal MPH exposure are unknown. Therefore, we exposed male and female Trinidadian guppies (*Poecilia reticulata*) to a low, chronic dose of MPH and observed that MPH affected the anxiety/exploratory behaviour of males, but not females. Because of this male-specific effect, we investigated the transgenerational effects of MPH through the paternal line. We observed behavioural effects of paternal MPH exposure on offspring and great-grandoffspring that were not directly administered the drug, making this the first study to demonstrate that paternal MPH exposure can affect descendants. These effects were not due to differential mortality or fecundity between control and MPH lines. These results highlight the transgenerational potential of MPH.

## Introduction

The parental environment can have a strong influence on offspring development and subsequent phenotype^1^. Evidence is now emerging that these parental effects can be transgenerational, i.e. spanning several generations; for example, stressful situations (e.g. early life stress)^2,3^, and exposure to some drugs and pollutants can have transgenerational effects on multiple traits including physiology and behaviour, e.g.^4–13^. If these effects are common, they would have important implications for understanding how natural populations respond to changing environments, including anthropogenic effects (e.g. climate change, exposure to pollutants) and how the experiences of our ancestors can affect our health and susceptibility to disease^14^. However, it is not clear if these results are generalizable beyond the few taxa where these effects have been investigated. In this study, we examine the transgenerational effects of a common pharmaceutical on natural behaviours of Trinidadian guppies, a freshwater, tropical fish.

Parental effects can be transmitted by the mother and/or father. It is well established that the maternal environment can affect offspring phenotype in both adaptive^1^ and nonadaptive ways^15^. In contrast, paternal effects, the influence of fathers on their offspring by nongenetic means, have received much less attention because, unlike maternal effects, there was no clear mechanism for their effects, except in species where males provide parental care or resources to females^16^. Recent evidence suggests that nongenetic factors, such as compounds in ejaculate and epigenetic/cytoplasmic modifications to sperm, can affect offspring phenotype^16,17^. Fathers can have additional effects on their offspring through their interactions with females, for example, when females differentially allocate resources to offspring based on the quality of their mate^16–18^.

Evidence is accumulating that parental effects can extend beyond offspring, influencing subsequent generations. Maternal effects can span multiple generations, affecting progeny that were not directly exposed to the original stimulus: ‘grandmaternal effects’ in externally fertilizing species^19–21^, and ‘great-grandmaternal effects’ in internally fertilizing species^22–27^. There is some evidence that paternal effects can extend beyond the offspring generation. These studies have demonstrated that stressors, including fear conditioning^28^, maternal separation^3^, restraint^29^, obesity^30–32^, drug exposure^4^, and exposure to pollutants^5,6^ are associated with altered phenotypes that span multiple generations through the paternal line. However, the conclusions that can be drawn from these studies may be limited for several reasons. First, many of these studies do not report potentially confounding effects of differences in survival or reproductive success between control and treatment lines. Second, some studies only investigate the effects in progeny of one sex, despite evidence that parental effects are sometimes sex-specific^33–37^. Third, most examples come from rodents (but see^5,14,27,37^), which makes it unclear if these results can be generalized to other taxa.

For our study of transgenerational effects, we manipulated the dopaminergic pathway with a pharmaceutical for several reasons. Dopamine is a neurotransmitter that is conserved across vertebrates^38^ and is involved in a variety of behaviours that are ecologically relevant in natural populations, such as sexual motivation^39,40^ and novelty-seeking behaviour^41,42^. Novelty-seeking behaviour can have fitness consequences for individuals in natural populations, including increased access to novel food sources, mating opportunities, and suitable habitats^41,43^, but at the cost of increased risk of predation^43^. We also focused on dopamine because it has been postulated that some drugs that affect the dopaminergic system will cause transgenerational effects^44^ and some of those drugs are widely used in humans.

In this study, we manipulated Methylphenidate hydrochloride (MPH), commercially known as Ritalin (Novartis) or Concerta (Janssen Pharmaceuticals Inc.). It is a stimulant that affects the dopaminergic pathway and was predicted to have transgenerational effects^44^. MPH provides therapeutic benefits by binding dopamine transporters on the plasma membrane of presynaptic neurons; this blocks the reuptake of this neurotransmitter (as well as norepinephrine), resulting in an increase in synaptic dopamine^45,46^. MPH is widely prescribed to individuals with Attention-deficit/hyperactivity disorder (ADHD^47–50^); this disorder is characterized by imbalances in the dopaminergic and noradrenergic systems of the brain, which results in reduced focus and increased impulsivity/over-activity. Further, ADHD is often comorbid with other psychological disorders and/or drug abuse and, therefore, can have detrimental effects on quality of life if left untreated^51^.

Despite its widespread use, the transgenerational effects of MPH are relatively unexplored. Studies in lab rodents and zebrafish have shown that maternal exposure to MPH can affect the neurochemistry and behaviour of offspring^52–55^. Further, it has been suggested that MPH can affect the male germline, as male rodents exposed to MPH through adolescence and into early adulthood (postnatal days 21 to 60) exhibited altered sperm morphology and germ cell epithelium structure^56^. However, the effects of paternal MPH on offspring and later generations in any species are currently unknown, and we are not aware of any transgenerational epidemiological studies on the effects of MPH in humans. This is surprising, in light of the high prescription rates to adolescent males (> 7%^49^), and the fact that similar drugs (e.g. cocaine) can cause paternal effects^57–61^. Specifically, MPH shares some neurochemical properties with cocaine and, in rodents, repeated exposure to either MPH or cocaine can lead to an increased prevalence of stereotypical behaviours, drug-seeking, and affective disorders, although some of these effects are only observed at high doses of MPH^62^.

In this study, we investigated if MPH causes transgenerational effects in Trinidadian guppies, *Poecilia reticulata*. Guppies and other fish, in general, are increasingly being used for neurological research because of their similarity to mammals in patterns of neurodevelopment, functional brain organization, and neurocircuitry, including the dopaminergic system^63,64^. In addition, female guppies carry eggs internally for approximately 25 days^65^ with small amounts of maternal transfer^66^. Further, novelty-seeking behaviour is ecologically relevant for wild guppies: guppies are highly attracted to novel stimuli^67–70^, and exploration and subsequent dispersal can increase mating success in both sexes^71,72^. Finally, previous studies have demonstrated that responses by guppies to MPH are similar to those observed in mammals^73^.

For this study, we administered MPH to the first-generation (G_1_) male and female guppies and asked if there were effects on their behaviour, and on the behaviour and whole brain dopamine levels of three subsequent generations that were not administered the drug directly. Because chronic treatment with therapeutic levels of MPH reduced rodent exploration^74^ and increased anxiety^74,75^, we predicted that guppies chronically treated with MPH would reduce their exploration of a novel environment. Indeed, we did observe a significant effect of chronic MPH on the exploratory behaviour of first generation (G_1_) males, but not females, so we subsequently focused on the transgenerational paternal effects of MPH exposure. Because paternal exposure to cocaine increased anxiety in rodent offspring^60^, we also predicted that offspring of MPH treated male guppies would exhibit increased anxiety, and therefore reduced exploration, as adults. However, we were unable to predict the direction of the behavioural responses of later generations (i.e. grandoffspring, great-grandoffspring) as there is not a consistent pattern in the literature about the direction or strength of induced phenotypes transmitted across multiple generations^76^. Finally, for transgenerational effects on brain dopamine levels, Lepetiller et al.^55^ observed that semi-chronic, prenatal MPH exposure increased dopamine levels in adult male rodents, so we predicted that progeny would also have increased dopamine levels relative to controls.

## Results

### Effects of chronic MPH administration

For the first step of this project, first generation (G_1_) juvenile guppies assigned to the MPH treatment began receiving a low, chronic dose of MPH (2.5 × 10^–8^ g/mL) from one month of age until testing, while Control individuals were treated in the same manner, except that they were only administered the vehicle. To mimic the therapeutic use of this drug in humans^47^, we treated guppies chronically by administering MPH three days/week to ensure that levels remained relatively stable in the aquarium water. Adult guppies were tested in the open field test, a standardized test that is widely used to measure locomotion, anxiety, and exploratory behaviour^77^. On average, we tested seven G_1_ individuals per brood (mean: 6.8, min: 1, max: 12) from one to three broods per pair (mean: 1.6), with half belonging to the MPH treatment group and the other half belonging to the Control group. We summarized recorded behaviours (freezing, ‘cautious swimming’ (i.e. slow swimming without movement of caudal fin), ‘wall-running’ (i.e. thigmotaxis), swimming, and duration located in the central area (Table S1)) with a Correspondence Analysis (CA): an ordination method conceptually similar to PCA^78^. We analyzed the first two components of CA (CA1 and CA2) using linear mixed effects models.

**Figure 1:**
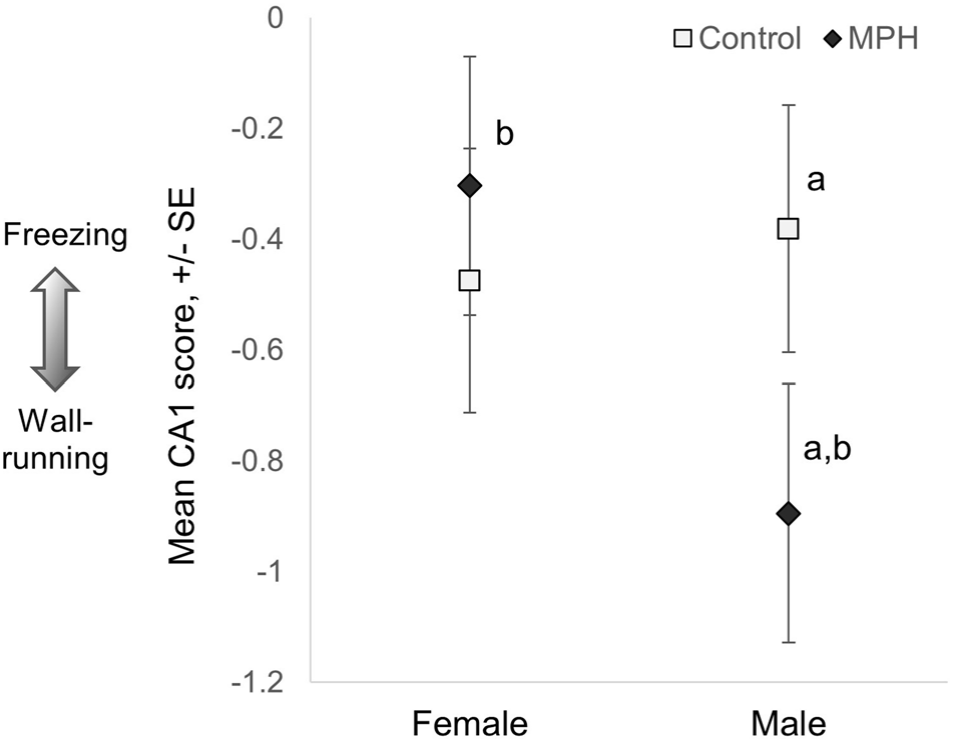
First generation (G_1_) CA1 scores for the open field test. MPH treated males performed significantly more wall-running than Control males and MPH treated females, and there was a trend for them to perform more wall-running than Control females. Positive CA1 values indicate relatively more freezing and negative values indicate relatively more wall-running. Symbols represent least-square means for each response variable, +/− one standard error. Letters indicate points that are significantly different from one another at *P* < 0.05.

For CA1, cautious behaviours (freezing and cautious swimming) loaded in the opposite direction of ‘wall-running’ (CA1 loadings: freezing: 1.57, cautious swimming: 1.26, inner squares: 0.75, swimming: − 0.19, wall-running: − 1.68; biplot in Fig. S1). As predicted, G_1_ male guppies chronically treated with MPH exhibited increased ‘wall-running’ (Table 1; Fig. 1; n = 188; Table S2), which we interpret as anxiety/avoidance in response to a novel environment^73^. In contrast, Control males froze more; freezing is involved in predator evasion^79^ (Table 1; Fig. 1). This suggests that MPH treatment disrupted typical anti-predator responses to a novel environment^53,80,81^. However, we did not observe a significant effect of MPH treatment on female behaviour (post hoc tests in Table S3). There was not a significant effect of MPH treatment on the behavioural variation summarized by CA2 (Table 1).

**Table 1:** Final results of mixed model analyses of G_1_–G_4_ open field trials for CA1 and CA2.

Generation	Original model	Response	Final model	Estimate	DF_num_	DF_den_	*F* value	*P* value
G_1_	Treatment * sex * age, date tested, handling, time, drug administration	CA1	Intercept	− 0.892		74.1		0.0003
			Treatment	0.5134	1	166	1.79	0.18
			Sex	0.5911	1	170	3.68	0.057
			Treatment * sex	− **0.6847**	**1**	**175**	**6.62**	**0.011**
			Date	0.00219	1	178	2.9	0.09
		CA2	Intercept	0.08642		39.5		0.57
			Treatment	− 0.00959	1	168	0.01	0.94
			Sex	0.252	1	172	3.63	0.059
G_2_	G_1_ male treatment * sex * age, brood size, date tested, days until isolation, handling, time	CA1	Intercept	− 0.4604		45.3		0.0003
			G_1_ male treatment	− 0.0057	1	35.1	0.001	0.97
			Age	− 0.00346	1	272	5.37	0.02
			**G**_**1**_ male treatment * age	− **0.00307**	**1**	**230**	**5.11**	**0.025**
			Sex	0.3318	1	277	6.99	0.009
			Sex * age	0.00599	1	278	14.57	0.0002
			Time	0.00141	1	274	2.96	0.087
		CA2	Intercept	− 0.06745		70.7		0.6
			**G**_**1**_** male treatment**	**0.266**	**1**	**61**	**3.96**	**0.051**
			Age	0.00003	1	162	1.92	0.17
			Sex	0.07042	1	278	0.28	0.59
			Sex * age	− 0.00431	1	276	6.83	0.01
			Date	0.00421	1	151	9.26	0.003
G_3_	G_1_ male treatment * sex * age, brood size, date tested, days until isolation, handling, time	CA1	Intercept	− 0.3052		112		0.12
			G_1_ male treatment	0.07873	1	42.3	0.28	0.6
			Sex	− 0.08446	1	208	0.33	0.57
			Days until isolation	0.00135	1	188	3.69	0.056
			Handling	0.08132	1	205	11.23	0.001
		CA2	Intercept	− 0.6538		73.4		0.0003
			G_1_ male treatment	0.1478	1	75.4	0.73	0.4
			Sex	0.6919	1	201	17.96	< 0.0001
			Age	− 0.00552	1	203	10.27	0.002
			Sex * age	0.00415	1	203	4.3	0.04
G_4_	G_1_ male treatment * sex * age, brood size, date tested, days until isolation, handling, time	CA1	Intercept	− 0.442		38.2		0.026
			G_1_ male treatment	0.1786	1	27.3	0.37	0.55
			Sex	0.4158	1	155	4.17	0.043
			G_1_ male treatment * sex	− 0.1401	1	141	0.2	0.65
			Age	− 0.00514	1	58.8	0.04	0.85
			G_1_ male treatment * age	0.01121	1	92.9	0.39	0.53
			Sex * age	0.00859	1	157	0.001	0.99
			**G**_**1**_ male treatment*sex*age	− **0.01723**	**1**	**158**	**4.74**	**0.031**
			Date	− 0.00603	1	83.1	4.33	0.04
		CA2	Intercept	− 0.2083		30.8		0.02
			**G**_**1**_** male treatment**	**0.3839**	**1**	**49.7**	**5.55**	**0.022**
			Sex	0.09821	1	163	0.43	0.51

*Bolded text indicates that the model term ‘G_1_ male treatment’ or interactions involving this term are statistically significant at *P* < 0.05.

### Paternal effects of MPH

**Figure 2:**
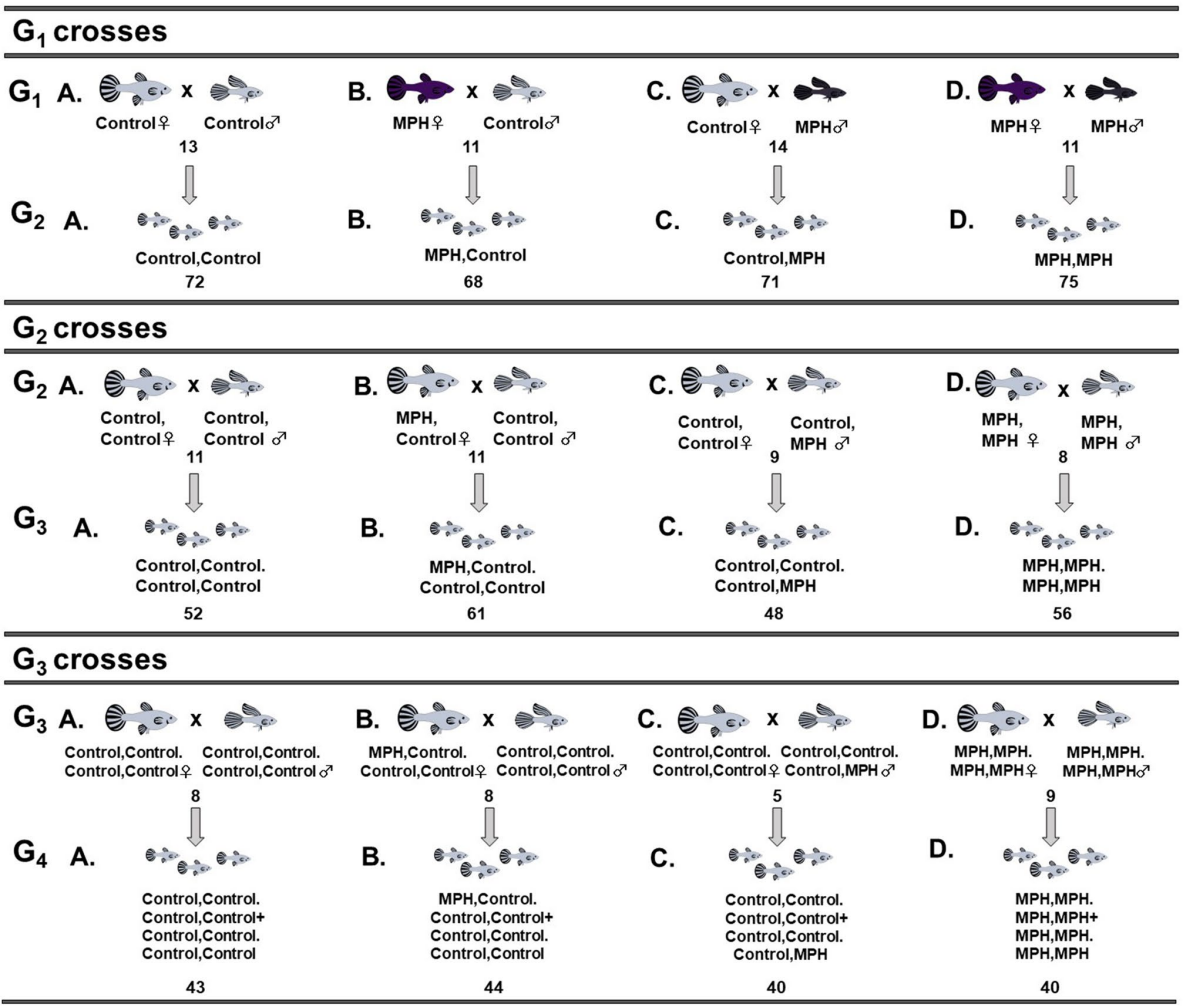
Crossing design for the G_2_-G_4_ generations. First-generation fish were mated using a factorial design to produce four offspring (G_2_) treatment groups: (**A**) Control female × Control male; (**B**) MPH treated female × Control male; (**C**) Control female × MPH treated male; and (**D**) MPH treated female × MPH treated male. These treatment lineages were maintained to produce the third (G_3_) and fourth (G_4_) generations. Because we saw an effect of MPH treatment on G_1_ males, but not G_1_ females, for statistical analyses of G_2_-G_4_ cohorts, we pooled the original treatment groups into two categories: progeny that were descendants of (i) G_1_ Control males (A. + B.) or (ii) G_1_ MPH treated males (C. + D.). Numbers underneath pairs of fish represent the number of unique lines that contributed to the following generation. Numbers underneath the corresponding progeny groups (small fish) represent the sample size for that group in the open field test. In total, we measured the behaviour of 858 fish. Dark shading in G_1_ fish represents MPH treatment.

To investigate the transgenerational effects of MPH treatment, first-generation fish were mated using a factorial design to produce four offspring (G_2_) treatment groups, which were maintained to produce the third (G_3_) and fourth (G_4_) generations (Fig. 2). The G_2_–G_4_ cohorts were not administered MPH or vehicle. For G_2_–G_4_ cohorts, we tested an average of three offspring per brood (G_2_ mean: 3.3, min: 1, max: 11; G_3_ mean: 2.7, min: 1, max: 8; G_4_ mean: 2.7, min: 1, max: 8) from one to five broods per pair (G_2_ mean: 1.8, min: 1, max: 4; G_3_ mean: 2, min: 1, max: 5; G_4_ mean: 2.1, min: 1, max: 5).

**Figure 3:**
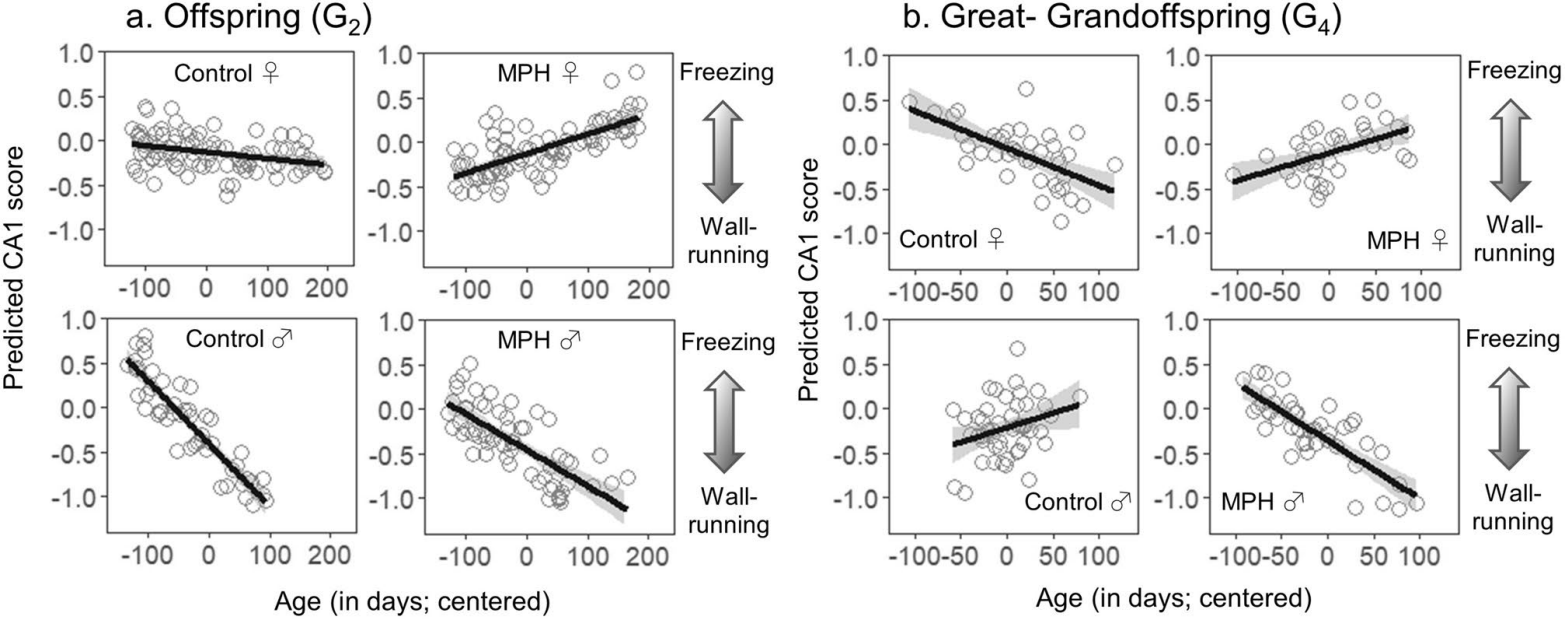
Associations between age and CA1 scores in the open field test for offspring (G_2_; panel **a**) and great-grandoffspring (G_4_; panel **b**). Panel (**a**): Male offspring of both Control (‘Control ♂’) and MPH treated (‘MPH ♂’) sires that were tested at a younger age froze relatively more than individuals tested at older ages. For female offspring of Control sires (‘Control ♀’), there was not an association between age and CA1 score but, in contrast, female offspring of MPH treated sires (‘MPH ♀’) froze more if tested at a relatively older age. Panel (**b**): All groups for the great-grandoffspring (G_4_) cohort exhibited qualitatively similar patterns to the same groups from the G_2_ cohort, except for male great-grandoffspring descended from G_1_ Control males (‘Control ♂’); however, this difference was not statistically significant. Positive CA1 values indicate relatively more freezing and negative values indicate relatively more wall-running. Lines were fit to the predicted values (which incorporate model estimates and random effects) using ‘lm’ in R and shading corresponds to the 95% confidence intervals.

There was not a significant effect of G_1_ female treatment on the behaviour of any progeny generation (G_2_–G_4_), so we focus on the effects of G_1_ male treatment. Note that analyses that retained the distinction between G_1_ female and G_1_ male treatment groups produced very similar results to the results presented here (see Table S4). Offspring of MPH treated fathers and Control fathers (hereafter called ‘MPH treated’ and ‘Control’ offspring for brevity) differed in the behaviours summarized by both CA1 and CA2. Similar to their parents, for CA1, there were sex-specific effects of MPH but, this time, female offspring differed based on their fathers’ treatment status (Table 1; Fig. 3A; n = 286). Female behaviour depended on their fathers’ treatment and the age at which they were tested: the behaviour of ‘Control’ females did not change significantly with age, but ‘MPH treated’ female offspring that were tested at a relatively old age froze more than younger individuals (slopes in Table S5). In contrast, ‘MPH treated’ and ‘Control’ males did not differ in how their behaviour changed with age.

**Figure 4:**
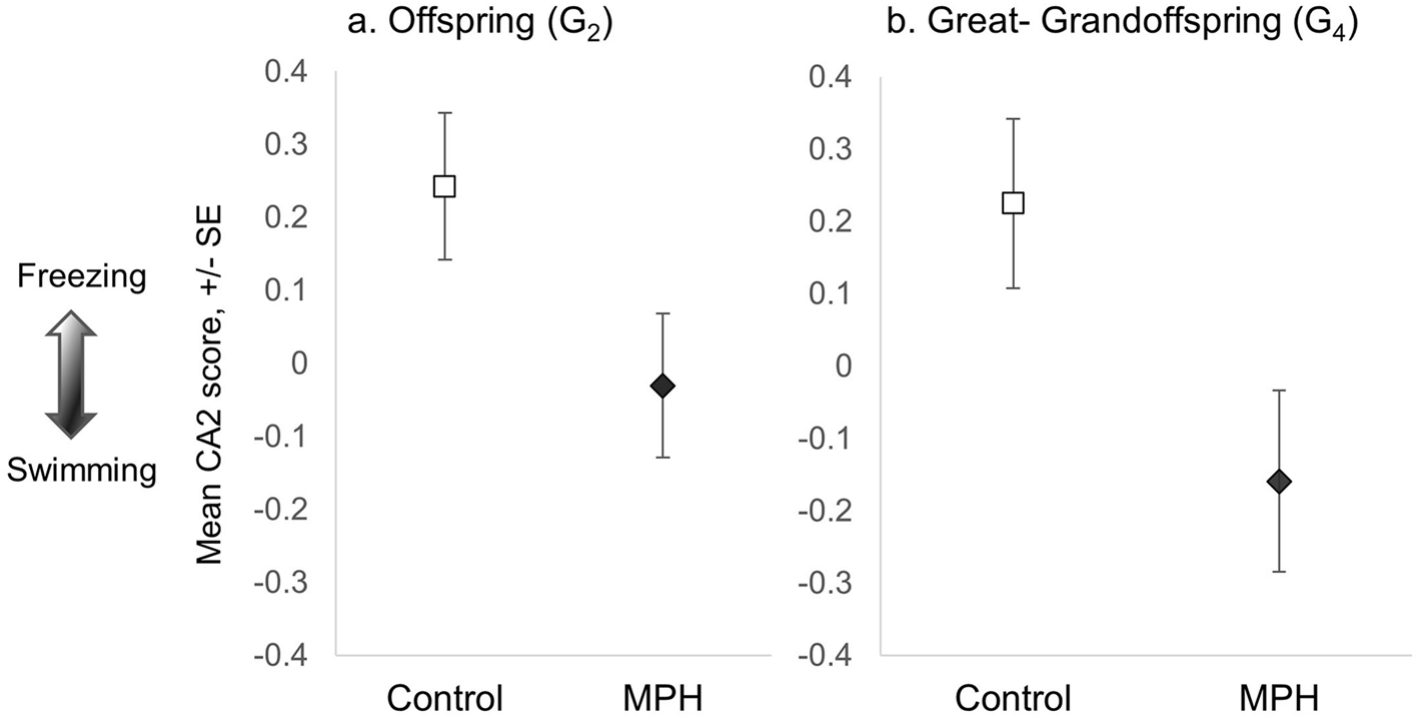
CA2 scores for offspring (G_2_; panel **a**) and great-grandoffspring (G_4_; panel **b**) in the open field test. Panel (**a**): Offspring of MPH treated sires spent significantly more time swimming in the open field tub than offspring of Control sires. Panel (**b**): Great-grandoffspring (G_4_) descended from G_1_ MPH treated males spent significantly more time swimming throughout the open field tub than great-grandoffspring descended from G_1_ Control males. Positive CA2 values indicate relatively more freezing and negative values indicate relatively more swimming. Symbols represent least-square means for each response variable, + /− one standard error.

For CA2, cautious behaviours loaded in the opposite direction of swimming and duration in the center of the arena (representing exploration or boldness^82^), so this axis represents a continuum between shyness/avoidance and boldness/exploration in response to a novel environment (CA2 loadings: freezing: 2.07, wall-running: 1.14, cautious swimming: 0.71, inner squares: -0.57, swimming: − 0.77). As predicted, ‘MPH treated’ offspring again differed from ‘Control’ offspring (Table 1; Fig. 4A; n = 286), with ‘Control’ offspring freezing more than ‘MPH treated’ offspring. This suggests that ‘MPH treated’ offspring were less cautious when investigating a novel environment. There was not a significant difference between male and female offspring. Taken together, results for CA1 and CA2 suggest that G_1_ male MPH treatment affected offspring response to a novel environment, with a stronger effect on the anxiety/avoidance behaviours of their daughters than their sons.

### Transgenerational effects of MPH

To determine if these effects were transgenerational, we also tested the behaviour of ‘Control’ and ‘MPH treated’ grandoffspring (G_3_) and great-grandoffspring (G_4_). There was not a significant effect of G_1_ male treatment on grandoffspring (G_3_) behaviour (Table 1; n = 213). Due to logistical constraints, the median ages for G_3_ males and females were higher than for the other generations and this may have influenced the results for this generation (Female median age (days): G_1_ = 132, G_2_ = 267, G_3_ = 394, G_4_ = 269; Male median age: G_1_ = 117, G_2_ = 231, G_3_ = 300, G_4_ = 248; Table S6 Figs. S2–S3). However, for great-grandoffspring (G_4_) behaviour, similar to the pattern we observed for the G_2_ (offspring), there were significant effects of G_1_ male treatment on both CA1 and CA2 (Table 1; n = 167; CA1: Fig. 3B; CA2: Fig. 4B). All great-grandoffspring treatment groups exhibited behavioural patterns similar to their G_2_ ancestors with one exception: the association between age and CA1 score appeared to differ for G_2_ and G_4_ ‘Control’ males (lower left panels, Fig. 3A,B). To determine if this apparent difference was statistically significant, we performed an additional analysis comparing only G_2_ and G_4_ ‘Control’ males, including the interaction between ‘generation’ and the covariate age. This analysis revealed that the CA1 scores for G_2_ and G_4_ ‘Control’ males did not significantly differ (*F*_1,6.85_ = 0.02; *P* = 0.9; n = 97; Table S7, suggesting that behavioural patterns were consistent for both generations.

While there is no evidence to suggest that MPH has mutagenic or clastogenic properties^83^, it is possible that our behavioural results were due to differential survival or reproduction in ‘Control’ and ‘MPH treated’ lineages during the course of the experiment. To examine this possibility, we performed several analyses that we describe briefly here (details in SI Methods). To determine if survival differed between ‘Control’ and ‘MPH treatment’ individuals for each of the four cohorts (G_1_–G_4_ cohorts), we performed separate survival analyses, by sex, in SAS using *Proc Lifetest*, which uses the Kaplan–Meier estimator^84^. There was not a significant difference between ‘Control’ and ‘MPH treatment’ group survival curves for either sex for any of the four generations (Table S8; Figs. S4–S5). Further, there was not a significant difference between ‘Control’ and ‘MPH treated’ groups in their fertility (whether or not they produced offspring; Table S9), fecundity (number of offspring produced; Table S10), or number of broods produced (Table S11). Finally, to determine if MPH treatment affected offspring sex ratio, we asked if the sex ratio of ‘Control’ and ‘MPH’ groups deviated from expected (1:1), and if the proportion of offspring that were male per pair (number male offspring/total offspring produced by a given pair) differed for ‘Control’ and ‘MPH’ groups for the G_2_–G_4_ cohorts. There was not a significant effect of MPH treatment on sex ratio (Table S12) nor on the inter-pair variation in proportion of offspring that were male (Table S13). Taken together, these results suggest that the effects of these factors on the behavioural differences between ‘Control’ and ‘MPH treated’ lines were minimal.

Because MPH functions by regulating the dopaminergic pathway^45,46^, we measured whole brain dopamine concentration for a subset of individuals from the G_2_ to G_4_ cohorts immediately following behavioural testing (SI Methods for detail). Contrary to our prediction, there was not a significant effect of G_1_ male treatment on whole brain dopamine concentration for any generation tested (G_2_ to G_4_, all *P* > 0.5; Table S15).

## Discussion

The main aim of this study was to determine if parental exposure to MPH, a drug commonly prescribed for ADHD treatment, leads to transgenerational effects in generations that were not administered the drug directly. We exposed G_1_ male and female guppies to a chronic, low dose of MPH, which affected male behaviour in the open field test. The offspring and great-grandoffspring of MPH treated males also exhibited altered behaviour relative to controls, demonstrating that MPH can cause transgenerational effects through the paternal line. Below, we discuss possible explanations for the lack of a significant effect of MPH on G_1_ females and G_3_ fish. Further, MPH treatment did not have a significant effect on whole brain dopamine levels, suggesting that the behavioural differences in progeny were not directly modulated by altered dopamine levels in the brain. Because fish and mammals share functional similarities in their brain neurochemistry and behaviour^63,64^, we suggest that our results may be relevant for mammals, including male adolescents and adults who are prescribed MPH.

The behavioural results for G_1_ males are similar to those observed in rodents that were chronically administered MPH^74,75^. One way to interpret our results is to consider behavioural patterns that would be adaptive in in the wild: freezing in response to an unfamiliar environment allows individuals to assess potential threats and reduce detection by predators^80,81^. G_1_ Control males froze in response to the novel environment, which suggests that the reduced freezing and increased ‘wall-running’ of G_1_ MPH treated males (and their offspring and great-grandoffspring) would not be adaptive in the wild. Zebrafish exposed to MPH during development also showed reduced freezing in a novel environment^53^. Further, our results for female progeny were age-dependent with the female descendants of G_1_ MPH treated males freezing more when they were older (and therefore, larger) when tested. Because predation risk declines with size in the source population used for this experiment, selection pressure on larger females to freeze should be relaxed^85–87^, suggesting that ancestral exposure to MPH interfered with this adaptive response. An exciting next step will be to test if environmentally relevant levels of MPH^88,89^ can also lead to transgenerational effects.

While MPH treatment caused a significant effect in G_1_ males but not G_1_ females, this sex-specific effect was reversed in their progeny for one behavioural metric (CA1), with only female offspring and great-grandoffspring differing in behaviour based on their father’s/great-grandfather’s treatment. Other studies have observed transgenerational effects expressed in the sex opposite to the one originally affected by an environmental stimulus^33–37^. This result further emphasizes the importance of studying transgenerational effects in both sexes.

Determining how MPH caused paternal effects was not part of this study, but it is possible that the effects on offspring resulted from nongenetic modifications to sperm/ejaculate and/or female-mediated paternal effects, both of which would be relevant in natural populations^16,17^. In rodents, MPH alters sperm morphology and germ cell epithelium structure^56^, and drugs that are neurochemically similar to MPH (e.g. cocaine) can cause epigenetic modifications to sperm with subsequent effects on offspring behaviour^59^. To determine the relative contributions of nongenetic epigenetic effects and female-mediated paternal effects in studies of paternal effects, future studies could use artificial insemination to tease apart the effects of male behaviour on female preference/physiology and paternal nongenetic effects.

An important finding of our study is the evidence for transgenerational effects in great-grandoffspring, a generation that was not directly exposed to MPH. Epigenetic modifications have been implicated as a mechanism for transgenerational effects in rodents (although this is contentious^90,91^). Mammalian embryos and germlines undergo epigenetic reprogramming (e.g. erasure of methylation marks), but it has been proposed that epigenetic marks can resist this reprogramming if the environmental stimulus occurs during a critical period^92^. The G_1_ male guppies in our study began receiving MPH at one month of age, so their germ cells were exposed to MPH during two critical periods: (i) prior to ‘puberty’ (when germ cells differentiate into spermatogonia), and (ii) during spermatogenesis^92^. In medaka (*Oryzias latipes*), a fish relatively closely related to guppies^93^, epigenetic reprogramming mechanisms are similar to rodents^94,95^. Therefore, if guppies have reprogramming mechanisms like medaka’s, it is possible that epigenetic mechanisms may be responsible for our transgenerational results; future studies could use in vitro fertilization to disentangle the effects of epigenetic modifications to sperm and other potential factors including the effects of male behaviour on female physiology or provisioning to offspring^16,17^. Further, because guppies have internally developing embryos, we suggest that guppies could be a ‘natural’ comparator for investigating transgenerational mechanisms in live bearing species.

We observed significant effects of G_1_ male MPH treatment on offspring (G_2_) and great-grandoffspring (G_4_) behaviour, but not grandoffspring (G_3_) behaviour. There are at least two explanations for this pattern: (i) we may have missed the effect in the G_3_ because of logistical issues with running this large experiment, and (ii) the expression of behaviour skipped a generation. We may have missed the ‘window of effect’ for the G_3_ because many individuals of this generation were tested at an older age than other generations (significantly higher median age). Age had a significant impact on the behaviour of G_2_ and G_4_ guppies, and rodent behaviour in the open field test also changes with increasing adult age^77,96–98^. Alternatively, it may be that the effect of MPH does disappear for a generation to return in the next. While other studies have observed the disappearance of a phenotype in one generation with a subsequent return in the following generation^99,100^, the mechanism for this is unclear.

Surprisingly, we did not see significant effects of MPH administration on G_1_ females nor on their offspring exposed in utero*,* i.e. there was not a significant effect of G_1_ female treatment on the behaviour of their offspring (or on any other generation tested). One possible explanation for this is that mature female guppies become significantly larger than mature males and, thus, females may have taken up a relatively lower amount of MPH per mg body mass from the water in their aquarium (doses were not adjusted for body size). However, given the significant effects of an acute dose of the same concentration on female guppies in the open field test^73^, this explanation seems unlikely. Alternatively, given the sex-specific differences in the dopaminergic pathway^101,102^ and other aspects of their biology, it is possible that chronic MPH administration had different effects on males and females.

In this study, we used individuals from an outbred population to investigate transgenerational effects. Therefore, one caveat is that we cannot rule out the possibility that genetic differences between MPH and control lines contributed to our results, however, this issue is not unique to this study^103–106^. We did attempt to standardize initial variation between Control and MPH lines by assigning full siblings to both treatments, and we determined that MPH treatment and Control lines did not significantly differ in their mortality, fecundity, fertility, offspring sex ratio or number of broods produced.

We measured dopamine levels in progeny generations (G_2_ to G_4_ cohorts) because MPH functions by increasing available dopamine^45,46^, and because rodents prenatally exposed to MPH had increased dopamine levels as adults^55^. However, we did not observe a significant association between paternal MPH treatment and progeny dopamine concentration. Although this is speculative, one possible explanation for why we observed behavioural effects of MPH across generations, but did not see the accompanying effect on dopamine levels, is that chronic G_1_ male MPH treatment affected the expression of genes related to other neurotransmitter pathways (e.g. serotonin, glutamate), and this altered expression was transmitted across generations. Other studies have shown that dopamine levels returned to baselines levels after chronic MPH use has ceased but, despite this, altered behavioural patterns persisted; those studies suggested that other neuroadaptations were responsible for these persistent behavioural changes^53,107^. In rats, chronic MPH use during adolescence is associated with persistent changes in the expression of genes involved with glutamate and serotonin receptors, and these have been linked to reward dysfunction, reduced impulsivity, and perseverative behaviour^108^. An important next step would be to determine if chronic exposure to MPH affects the expression of genes involved with glutamate and serotonin receptors.

As far as we are aware, this is the first study of any species to demonstrate that paternal exposure to low, clinically relevant levels of MPH during adolescence and early adulthood can affect offspring phenotype. Because of the similarities between fish and mammals in the dopaminergic system^63^ and in behavioural responses to MPH^53,73^, our results are likely relevant to humans. This could have widespread implications given the relatively high prescription rates of MPH to males^49,50^, and because there are currently no precautions for MPH use for men planning to have children^109,110^. Because drug-seeking behaviour is associated with novelty-seeking^41^, offspring of men taking MPH could be prone to an increased propensity for drug abuse^41^ and other affective disorders^51^. The next step will be to verify our results in mammalian models.

This study contributes to the accumulating evidence that paternal effects have the potential to span multiple generations. Further, because the behavioural responses of guppies to acute^73^ and chronic doses of MPH resemble behavioural responses in mammals, we suggest that the Trinidadian guppy is an excellent comparator for studying the effects of drugs on behaviour and neurochemistry. Future studies should continue to explore transgenerational effects in other natural systems to determine the generality of these effects.

## Methods

To produce the first generation (G_1_), virgin females were each housed with one male. Thus, all offspring of a given pair were full siblings and were part of the same ‘lineage’ (Fig. 2). For up to two broods per female, offspring were moved into sibling tanks in the same experimental chamber within 24 h of birth. At one month of age, all focal individuals were moved to their own tank for treatment. All focal fish were housed with an unrelated, non-focal juvenile to avoid the stress of social isolation (details in SI Methods).

### MPH administration

Treatment with MPH (or vehicle) began at one month of age (30 days old) because this roughly corresponds to human adolescence (~ 12–18 years old), a period when neurotransmitter systems, including the dopaminergic system, undergo maturation and rearrangement^111^. Based on gonopodium development^112^, the male guppies in our study became sexually mature at approximately 60 days after birth (median: 60 days; range: 42–74 days) and began exhibiting sexual behaviour one to two weeks before maturity^112^ (note: there was not a significant effect of MPH treatment on age at sexual maturity (*F*_1,51.4_ = 0.45; *P* = 0.5)). Female guppies become sexually mature at approximately the same time as males^112^.

An acute, low dose of MPH (2.5 × 10^–8^ g/mL) affected female guppies’ behaviour in a novel environment^73^ so we used the same dose for chronic treatment in this study. We administered MPH to treatment individuals every other day, as high-performance liquid chromatography showed that this drug delivery schedule maintained aquarium MPH levels at approximately 2.5 × 10^–8^ g/mL. We dissolved MPH in dechlorinated water before administering doses to treatment tanks with a syringe. Control individuals received water, without MPH, with a syringe. Because we wanted to ensure that MPH concentration in treatment tanks remained relatively consistent over the course of our study and because the densities of guppies were low, we did not perform partial water changes on any experimental tanks. All tanks were covered with fitted lids to reduce water evaporation and, once per week, water was topped up to the original volume. To ensure tanks remained clean and aerated, each tank was equipped with an airstone and ~ three snails. When tanks needed to be cleaned (~ once every two months), the fish and tank water were gently added to another container, the tank was thoroughly cleaned, and then the original water was poured back into the tank. The same cleaning procedures were used for MPH treatment and Control tanks. The G_2_–G_4_ cohorts were not administered MPH or vehicle, but tank cleaning and maintenance was performed in the same way as for the G_1_ cohort.

### Behavioural assays

We assessed novelty-seeking behaviour of guppies using the widely used open field test, which assesses the behaviour of an individual placed in a novel, open environment^73,77,82,113^. The metrics used for this test can represent an interplay between curiosity/motivation to explore and fearfulness, and it has been validated for these behaviours in guppies^82^. Fish and the water from their ‘home tanks’ were moved to a new tank in the testing room at least four days before testing to allow them to acclimate to room conditions. On the day of testing, fish were fed at least 30 min prior to testing. For the G_1_ cohort only, if it was a ‘drug administration’ day, fish were given their respective treatment (MPH for MPH treatment fish or the vehicle for Controls) at least 30 min prior to testing by a research assistant who knew the treatment status of the fish. As in previous studies^73,82^, the open field test was conducted in a 33 × 28 × 12 cm green plastic tub, with black lines delimiting 5.5 × 7 cm rectangles on the bottom, containing 5L fresh conditioned water. Behavioural observations commenced 10–15 s after the experimental fish was gently netted into the tub to ensure that we captured the initial responses to the novel environment. Behaviours were recorded for seven minutes using JWatcher (version 1.0^114^). The observer (AD) was blind to treatment status when scoring behaviour. The water in the tub was replaced with 5L fresh conditioned water after each test. G_1_ tests were conducted between 9:00am to 1:00 pm, and this timeframe was further reduced to 9:00 am–11:30 am for the G_2_–G_4_ cohorts, because an assistant was no longer required to administer treatments. After testing, fish were either returned to their ‘home’ tank in the environmental room or were immediately prepared for brain dissection for dopamine quantification (SI Methods).

We summarized all behaviours in the open field test with a CA, except for the proportion of inner/total squares traversed (a metric of exploration^82,115,116^), as this behaviour was measured on a different scale than other behaviours. This behaviour was moderately correlated with CA1 and results were similar to those for CA1 (see Table S16 and Figs. S6–S7). We measured activity level (total squares traversed) because stimulant drugs, including MPH, can affect activity level independent of exploratory behaviour^117,118^; however, MPH treatment did not have a significant effect on activity (Table S17).

### Statistical analyses

We performed CA in R (version 3.4.4^119^) using the “corresp” function of the *MASS* package (version 7.3–49^120^). CA works well with data that are zero-inflated and that add to a fixed total^78^. Collectively, the first two components of the CA explained 77% of behavioural variation in the open field test (CA1: 49%; CA2: 28%; Fig. S1). We analyzed CA1, CA2 and other response variables with linear mixed effects models (*Proc Mixed*) in SAS (version 9.4^84^); all models met normality assumptions (based on residual plots). For the G_1_ cohort, we included treatment (MPH treated or control) and sex as main effects. We included age as a covariate, as behaviour in the open field test has been observed to change with increasing adult age^77,96–98^. Because MPH functions by affecting dopamine^45,46^ and because dopamine function can be sex and age dependent^121–123^, we anticipated that the behavioural effects of MPH could also be sex and age dependent. Therefore, we considered all interactions between MPH treatment, sex, and age. To account for variation in testing conditions, the following were included as additional covariates/cofactor: drug administration day (‘yes’ or ‘no’), time of day tested, date tested, and handling time (time to net the fish from their tank to the open field tub; see SI Methods). Guppy exploratory behaviour can be associated with body size^116^, and MPH can affect body size^124^, however, in this study, we did not observe that variation in body size (standard length) significantly contributed to the behavioural results (Tables S18–S19).We included parental lineage and brood identifier (because some females contributed multiple broods (we note that this happened at similar frequencies for females from each treatment)) as random effects. For analyses of the G_2_–G_4_ cohorts, we included G_1_ male treatment and sex as main effects and age as a covariate. We considered two additional covariates in these analyses: ‘days until isolation from siblings’ (because the number of days focal fish remained with siblings varied) and number of broodmates (see SI Methods for details). Because the large number of random and fixed effects created a risk of over parameterization, we only included biologically relevant interactions for which there was a priori justification; specifically, we considered all interactions among G_1_ male treatment, sex, and age, and removed non-significant interactions among those terms and other non-significant covariates in a stepwise fashion (*P* > 0.1).

Because individuals in the G_2_–G_4_ generations varied in how related they were to one another, we input the pedigree into *Proc Inbreed* to create a genetic covariance matrix. We included this covariance matrix in the model as a random effect^125^. In one case, a model with the full pedigree did not converge (G_4_ male dopamine analyses), so we removed this factor and instead included great-grandparental lineages and all relevant interactions as random effects (Table S15).

For all continuous covariates, we centered each variable about its mean^126^. We used the Kenward-Roger method to determine approximate degrees of freedom because sample sizes were not equal for each level of fixed effects^127^. When interactions between main effects were significant, post hoc comparisons between least-square means were performed using “simulate” in *Proc Mixed*.

In separate analyses of the G_1_, G_2_, and G_4_ generations, we observed significant effects of G_1_ male MPH treatment on behaviour. In total, 5 tests involving G_1_ male MPH treatment were significant at *P* < 0.05, whereas only 1.6 would be expected by chance (for each generation, 4 terms involved treatment for each of the 2 behaviours, for a total of 32 tests).

All procedures outlined in this study were approved by the Animal Care Committee at the University of Toronto (protocol numbers: 20008920, 20008921, 20009555, 20010160, 20010588, 20009045, 20010020, 20010527). Authorization to import and administer methylphenidate hydrochloride was obtained from the National Compliance and Exemption Division, Office of Controlled Substances, Health Canada (Authorization number: 26982.01.12). All experiments were performed in accordance with ARRIVE guidelines.

## Supplementary information

Supplementary Information 1.

Supplementary Information 2.

## Data Availability

All data generated and analyzed for this study are included in this published article (and its Supplementary Information files).
