# Supplementary material for: Paternal exposure to a common pharmaceutical (Ritalin) has transgenerational effects on the behaviour of Trinidadian guppies
Source: Sci Rep. 2021 Feb 17;11:3985. doi: 10.1038/s41598-021-83448-x (PMC7889922; doi:10.1038/s41598-021-83448-x)
Supplement: Supplementary file 2 — Supplementary Information 2. [file 41598_2021_83448_MOESM2_ESM.pdf]

Supplemental Information for:

Paternal exposure to a common pharmaceutical (Ritalin) has transgenerational effects on the behaviour of Trinidadian guppies

De Serrano, Alex R., Hughes, Kimberly A., Rodd, F. Helen

1. Department of Ecology and Evolutionary Biology, University of Toronto, 25 Willcocks St., Toronto, Ontario, Canada, M5S 3B2
2. Department of Biological Science, Florida State University, 319 Stadium Dr., Tallahassee, Florida, USA, 32304

*Corresponding authors, email: a.deserrano@utoronto.ca, helen.rodd@utoronto.ca

Table of contents for Supplemental Information

Supplemental Methods …………………………………………………………………..………….… 2

Supplemental Tables and Figures…………….…………………………………………………..… 9

Table S1 ……………………………………………………………………………...…….. 9

Figure S1 ………………………………………………………………………….…...…….. 10

Table S2 ……………………………………………………………………….....…….. 11

Table S3 ……………………………………………………………………….....…….. 12

Table S4 ……………………………………………………………………….....…….. 13

Table S5 ……………………………………………………………………….....…….. 16

Table S6 ……………………………………………………………………….....…….. 16

Figures S2 & S3 …………………………………………………..………….…......….. 17

Table S7 ……………………………………………………………………….....…….. 19

Figures S4 & S5 …………………………………………………..………….…......….. 20

Table S8 ……………………………………………………………………….....…….. 22

Table S9 ……………………………………………………………………….....…….. 23

Table S10 ……………………………………………..…………………….....…….. 23

Table S11 ……………………………………………..…………………….....…….. 24

Table S12 ……………………………………………..…………………….....…….. 25

Table S13 ……………………………………………..…………………….....…….. 26

Table S14 ……………………………………………..…………………….....…….. 28

Table S15 ……………………………………………..…………………….....…….. 28

Table S16 ……………………………………………..…………………….....…….. 30

Figures S6 & S7 …………………………………………………..………….…......….. 31

Table S17 ……………………………………………..…………………….....…….. 33

Table S18 ……………………………………………..…………………….....…….. 34

Table S19 ……………………………………………..…………………….....…….. 35

Supplemental References ………………………………………………………………….. 40

Supplemental Methods

Subjects and Housing

Experimental guppies were lab-reared descendants of fish from a low-predation tributary ('Houde' tributary (GPS: PS 896 886; N: 10.74740 61.26629)) of the Paria River in Trinidad [S1]. Fish were collected every few years from the field site and added to our stock tanks. This project was initiated in 2012; the most recent collection before this project began was 2008. All focal individuals from the G_1 to G_4 generations were reared in the same experimental chamber. The experimental chamber was illuminated with full spectrum fluorescent bulbs and was held in 12:12hr light:dark at 25-26˚C. Each focal fish was placed in its own 2-gallon tank (12.5 x 30 x 17 cm) containing 5L of water that had a layer of natural-coloured gravel on the bottom and a clump of algae for cover. Fish were fed twice daily: Tetramin fish food in the mornings, and live *A. nauplii* in the afternoons.

To produce the first generation (G_1), juvenile guppies (G_0) were taken from four large (75L) stock tanks, each with over 300 individuals, and were each isolated in a two-gallon tank with a smaller juvenile. G_1 broods were selected for inclusion in the experiment if a G_0 female's first brood consisted of at least six fish; this was done because fish were assigned to treatments before sexing was possible, so only using broods with 6+ fish improved the odds of having at least one male and one female sibling in both treatments (MPH treated and Control). G_1 focal individuals were placed in their own tank at one month of age and, at that time, drug or control treatment was assigned. To reduce the stress of social isolation, each focal fish was paired with a smaller, non-focal juvenile, haphazardly selected from stock tanks; to avoid confusion with the focal adult, these non-focals were replaced with younger juveniles as they began to reach sexual maturity. While collecting offspring that would contribute to the subsequent generation, females were checked daily after they were mated and, when new broods were discovered, offspring were transferred to a separate sibling tank. The G_2-G_4 cohorts were reared in the same manner as the G_1, except for the following differences: (i) they were not administered MPH or vehicle, (ii) individuals from broods with fewer than six fish were also included in the experiment, and (iii) individuals were often housed with their siblings for more than one month.

Dopamine ELISA details

Because MPH functions by regulating the dopaminergic pathway [S2,S3], we measured whole brain dopamine concentration for a subset of individuals from the G_2 to G_4 cohorts. For the G_2 cohort, only female brains were analyzed due to logistical constraints, but for the G_3 and G_4 cohorts, both male and female brains were measured. Immediately following behavioural testing (one or two minutes after testing), fish were sacrificed for whole brain dopamine quantification. Fish were rendered unconscious/dead by submerging them in an ice slurry for five to ten seconds; directly following this, fish were decapitated using a scalpel. Whole brains were removed from the skull and were placed in a drop of HCl-EDTA solution to preserve dopamine levels (BA 10–0300, Rocky Mountain Diagnostics kit instructions) and were kept on ice until all brains were collected that day (maximum four per day). Brains were weighed, and then added to 0.1 mL of HCl-EDTA. The brain tissue/HCl-EDTA solution was sonicated for 30 seconds, centrifuged for 30 minutes, and the supernatant was stored in a -20°C freezer until all samples for that generation were collected.

Dopamine concentration was determined using a Dopamine enzyme immune-assay kit (ELISA) following kit instructions (BA 10–0300, Rocky Mountain Diagnostics). Dopamine concentration was calculated from sample absorbances read at 450nm using a Biotek Synergy HT microplate reader with Gen5 software (v1.10.8; Biotek). We calculated the absorbance values of kit standards, and these values were used to create a standard curve (4-parameter curve equation) from which sample concentrations could be determined. The standards used to create the standard curve were: 0 ng/mL dopamine, 0.5 ng/mL, 1.5 ng/mL, 5 ng/mL, 20 ng/mL, and 80 ng/mL. The calculated concentration of each of these standards for each separate kit (we ran eight kits total) are included in Table S14.

Because our hypotheses were related to paternal transmission, for all statistical analyses, G_1 male treatment was included as a main effect. For the G_2 cohort, only females were measured, so age and brain mass were considered as covariates. For the G_3 and G_4 cohorts, both male and female dopamine levels were measured, and separate analyses were run for each sex as adult males and females differ in body size [S4,S5] and in brain size [S6]. Therefore, for each sex separately, G_1 male treatment was included as a main effect, and age and brain mass were considered as covariates.

Covariates in analyses

To account for variation in testing and rearing conditions, we considered several covariates in statistical analyses. Due to logistical constraints, there was variation in how long individuals in the G_2-G_4 cohorts were housed with siblings before they were isolated for behavioural assays (note: all individuals were isolated with a nonfocal juvenile for at least one month before testing.). Therefore, 'days until isolated' was included as a covariate in these behavioural analyses for the G_2-G_4 cohorts. As rearing density can affect behaviour in adult guppies [S7], we also included 'number of broodmates' as a cofactor in these analyses by creating bins for the number of fish in a brood (1 to 2, 3 to 6, 7+). Bins were created based on the tertiles (3-quantiles) of the data. We also included time of day tested (represented as minutes from 12:00 AM) and date tested (Julian date) as covariates. The amount of time it took to net the fish at the beginning of the trial (handling time) was an additional covariate, as this could affect stress levels. The majority of fish were 'netted' within 15s (856/860 were netted within 15s). We removed non-significant covariates from the final model if $P > 0.1$.

Analyses where G_1 female treatment was included

We did not include G_1 female treatment in the analyses of the G_2-G_4 cohorts in the main text because our hypotheses were related to the effects of G_1 male MPH treatment on the behaviour of progeny. We took this approach because there were no significant effects of MPH treatment on G_1 female behaviour (CA1 or CA2), however, it is possible that there were downstream effects of G_1 female MPH treatment on offspring. To determine if this was the case, in separate analyses (described here), we included G_1 female treatment as a main effect for analyses of behaviour of the individuals in the G_2, G_3, and G_4 cohorts. We included G_1 female treatment, G_1 male treatment, and sex as main effects, and age of the focal individual as a covariate. Additional covariates (time of day tested, date tested, handling time, days until isolation from siblings, and number of broodmates) and random effects (pedigree, brood) were incorporated as described in the main text. We considered all interactions among G_1 female treatment, G_1 male treatment, sex, and age, and removed non-significant interactions in a stepwise fashion ($P > 0.1$). Because G_1 female treatment was not involved as a main effect or in any significant interactions (Table S4), we conclude that G_1 female treatment did not have a significant effect on the behaviour of progeny generations (G_2-G_4) in the open field tests.

Survival analyses

For the G_1 cohort, the mortality dates for all individuals were known. For the G_2-G_4 cohorts, the dates of death of some non-focal individuals (i.e. siblings from large broods that were not used in the experiment) are unknown. However, the lack of information about these non-focal individuals should not bias the mortality results because there were non-focal individuals in all treatments. For all analyses, males and females were analyzed separately.

To determine if survival differed between Control and MPH treatment individuals for each of the four cohorts (G_1-G_4 cohorts) separately, we performed survival analyses, by sex, in SAS using *Proc Lifetest*, which uses the Kaplan-Meier estimator [S8]. For fish that were sacrificed for dissection, the age at which they were euthanized was entered as the death date, and this value was censored in the analysis.

Fertility and fecundity analyses

To determine if there was a difference in the fertility of MPH treated and control fish, we first asked if there was a treatment effect on whether or not focal fish produced offspring. To do this, we used Fisher's exact test, as it is more accurate than the chi-square test when sample sizes are small [S9]. Fish were only considered in this analysis if they had been paired with a member of the opposite sex. Some males were paired with two females; these males were scored as "Yes" if either of his mates produced offspring. We note that the number of pairs that did not produce offspring (i.e. "No") are higher for G_1 sires and dams than for G_2 and G_3 pairs. This is because G_1 fish were only paired with one individual, even if they did not produce offspring. Female guppies are choosy, and sometimes will not mate with the male provided. For G_2 and G_3 pairs, we wanted to ensure that there would be enough fish in the following generations for behavioural testing; therefore, if a pair did not produce offspring after two months, the male was replaced with his brother (when possible) or with another, unrelated male from the same treatment. This reduced the number of G_2 and G_3 sires and dams that did not produce offspring.

Second, for the focal fish that did produce offspring, we used Mann-Whitney tests to determine if there was a difference in the number of offspring produced by MPH treated and Control adults. Finally, for G_2 and G_3 pairs, we wanted to ensure that there would be enough fish in the following generations for behavioural testing. Thus, females that were used for breeding were not killed for dopamine analyses and were kept in the experimental chamber until they died

naturally; therefore, they often produced multiple broods. When females produced multiple broods, offspring from later broods (e.g. second and third broods) were also used for mating and behavioural testing. The number of broods in the 2+ category ranged from two to six broods, although most fish in this category produced two to three broods. We asked if fish from the two treatment groups varied in whether or not they produced multiple broods. To do this, we used the exact binomial test of goodness of fit [S9,S10].

Sex ratio analyses

To ask whether there was an effect of MPH treatment on offspring sex ratio, we first asked if the baseline sex ratio of G_1 offspring (i.e. offspring of G_0 dams) differed from expected, i.e. equal numbers of male and female offspring, using the exact binomial test [S9,S10]. We considered this a baseline as G_0 dams and sires were not exposed to any treatment (Control or MPH) and the sex ratio of the broods produced by these fish should represent the sex ratio of our lab population. We were also interested in determining if there was significant variation in the sex ratio of offspring across pairs. To investigate this, we analyzed the proportion of offspring that were male per pair (number male offspring/total offspring produced by a given pair) using *Proc GLIMMIX* with family lineage included as random effects, and intercept as the only explanatory variable [S8,S11]. The sex ratio of the G_1 cohort did not significantly deviate from 1:1 (Table S12) and there was not significant inter-pair variation (Supplemental Table S13; P = 0.65), i.e. the inter-pair variation did not significantly differ from random binomial variance. Therefore, we assumed an expected sex ratio of 1:1 for our analyses of the potential effects of MPH treatment on sex ratio for the G_2-G_4 cohorts.

To determine if the sex ratio differed from expected (1:1) for Control and MPH treated groups from the G_2-G_4 cohorts, we used Fisher's exact test. Inter-pair variation in offspring sex ratio was analyzed in the same manner as the G_1 cohort, except: (i) G_1 male treatment (Control or MPH treated) was included as the main effect, and (ii) parental lineages (and when applicable, grandparental and great-grandparental lineages) and their interaction were included as random effects for all analyses.

Analyses of inner/total squares traversed

As discussed in the main text, 'duration located in the central (inner) area' of the open field tub was included in the CA and, therefore, contributed to both CA1 and CA2 scores. Because duration spent in the inner squares (CA1 and CA2 scores) and movement through the inner squares (number of inner squares/total squares traversed) influenced all three behavioural metrics, we assessed if these behaviours were correlated. We used Pearson's correlation using the "cor.test" function in R [S12]. This revealed that inner/total squares traversed was moderately positively correlated with CA1 (corr = 0.66, $t_{858} = 25.6$; $P < 0.0001$) and moderately negatively correlated with CA2 (corr = -0.33, $t_{858} = -10.3$; $P < 0.0001$). Therefore, we did not discuss the results for inner/total squares in the main text but we include them here. For analyses of inner/total squares traversed, to ensure that residuals were normally distributed, this variable was square root transformed. Inner/total squares traversed was analyzed in the same way as were CA scores. For all analyses, the results for inner/total squares traversed were qualitatively similar to the results for CA1 (Table S16; Figs. S6-S7).

Behavioural analyses with body size as a covariate

In guppies, exploratory behaviour can vary in a size-specific manner [S13]; thus, to determine if the effects of MPH treatment on behaviour were size-dependent, we included body size (standard length) as a covariate in additional models. We do not include body size in the models discussed in the main text as we only recorded the body size of a subset of the individuals for which we had recorded behaviour, and we did not want to exclude observations for which we did not measure body size in the main analyses. Adult guppies exhibit sexual dimorphism in body size, so we ran separate analyses on males and females for each generation. For these analyses, G_1 male treatment was included as a main effect. Male guppies essentially stop growing at sexual maturity, so we did not expect a significant correlation between age and standard length for adult males. Indeed, there was not a significant association between age and body size for males from any generation (all $P > 0.1$; Table S18), so we include both age and body size as covariates in statistical models. For females, we investigated the correlation between age and body size to determine if these covariates were correlated. Age and body size were significantly correlated for G_3 females only (Table S18); therefore, for G_3 female analyses, we did not include female age in these analyses. For the remaining analyses for females, we

included both age and body size as covariates. Additional covariates (time, date, etc.) were included/excluded as described for the full behavioural analyses in the main text. In total, 2 tests involving standard length were significant at $P < 0.05$ (Table S19), whereas 5.2 would be expected by chance (for G_1, 4 terms involved standard length for each sex for each behaviour; for G_3 females, 4 terms involved standard length for each behaviour; and for the remaining cohorts (G_2, G_3 males, G_4), 8 terms involved standard length for each sex for each behaviour, totaling 104 tests). Thus, we observed fewer significant effects of body size than what would be expected by chance alone, so we conclude that variation in body size did not significantly contribute to the behavioural results in this study.

Supplementary Tables and Figures

Table S1. Descriptions of behaviours recorded during the open field trials that occurred frequently enough to be included in statistical analyses. All behavioural variables included in this table were summarized with a Correspondence Analysis.

Behaviour	Description	Interpretation	Additional notes	Adapted from
Cautious Swimming	Slow swimming behaviour, without use of caudal peduncle. Often occurs as the transition between freezing and swimming	Transition between fear/ shyness to more exploratory/ bold	Mutually exclusive from freezing, swimming, and wall-running	Pers. obs.
Freezing	Fish is not moving	Fearfulness. This is a common anti-predator behaviour	Mutually exclusive from cautious swimming, swimming, and wall-running	[S14,S15]
Inner duration	Duration spent in the central squares of the arena	Indicator of low anxiety or high exploration/ boldness	Mutually exclusive from wall-running	[S16,S17]
Swimming	Ambulatory behaviour; swimming with use of caudal peduncle	Indicator of low anxiety or high exploration/ boldness	Mutually exclusive from cautious swimming, freezing, and wall-running	[S14,S15]
'Wall-running'	Rapid swimming along the walls of the test arena	Stress response/ similar to thigmotaxis (wall-seeking) behaviour	Mutually exclusive from cautious swimming, freezing, swimming, and inner duration. If this behaviour occurs later in the trial, it could also represent the desire to escape a simple environment	[S14,S16,S18]

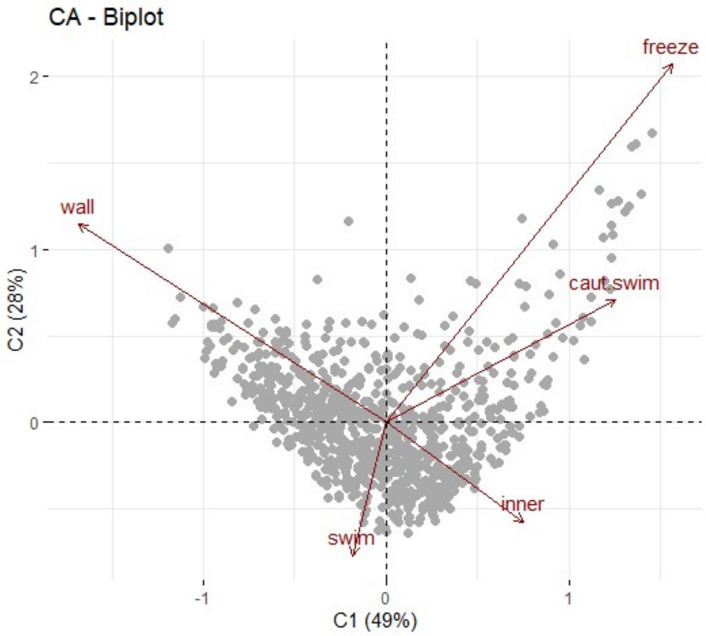

Variable	CA1 Loading	CA2 Loading
Cautious Swim	1.259	0.709
Freeze	1.569	2.068
Inner	0.752	-0.572
Swim	-0.187	-0.770
Wall	-1.684	1.143

Figure S1. CA biplot illustrating variation in behaviour in the open field test for all focal fish (G_1 to G_4; n = 858). Distance from the origin explains relative importance of that variable in explaining behavioural variation. This figure was created using the *FactoMineR* package in R (version 2.3 [S19]).

Table S2. The estimates for the random effects terms included in the models summarized in Table 1.

Generation	Behaviour	Final model	Covariance Parameter	Estimate	SE	*Z* value	*P* value
G_1	CA1	Treatment * sex, date	Line	0.1432	0.0765	1.87	0.031
			Brood	0	.	.	.
			Residual	0.7582	0.08361	9.07	< 0.0001
	CA2	Treatment * sex	Line	0.1805	0.09073	1.99	0.023
			Brood	0	.	.	.
			Residual	0.7934	0.08676	9.14	< 0.0001
G_2	CA1	G_1 male treatment * age, sex * age, time	Pedigree	-0.05446	0.03794	-1.44	0.15
			Brood	0.1093	0.06653	1.64	0.05
			Residual	0.8855	0.09646	9.18	< 0.0001
	CA2	G_1 male treatment, sex * age, date	Pedigree	0.0081	0.0653	0.12	0.9
			Brood	0.04938	0.0581	0.85	0.2
			Residual	0.9139	0.09549	9.57	< 0.0001
G_3	CA1	G_1 male treatment, sex, days until isolation, handling	Pedigree	0.03273	0.06291	0.52	0.6
			Brood	0.03542	0.0579	0.61	0.27
			Residual	0.8255	0.1081	7.64	< 0.0001
	CA2	G_1 male treatment, sex * age	Pedigree	0.04784	0.09276	0.52	0.61
			Brood	0.1673	0.08457	1.98	0.0239
			Residual	0.7195	0.09566	7.52	< 0.0001
G_4	CA1	G_1 male treatment * sex * age, date	Pedigree	0.1478	0.1525	0.97	0.33
			Brood	0.01738	0.1065	0.16	0.43
			Residual	0.7797	0.1389	5.61	< 0.0001
	CA2	G_1 male treatment, sex	Pedigree	0.0086	0.07853	0.11	0.91
			Brood	0.02887	0.07284	0.4	0.35
			Residual	0.8366	0.222	7.54	< 0.0001

Table S3. *Post hoc* comparisons for the significant Treatment*Sex treatment interaction for CA1, which was performed using "simulate" in *Proc Mixed*.

Generation	Behaviour	Comparison	Estimate	*df*	*t* value	Unadjusted *P* value	Adjusted *P* value*
G_1	CA1	Control female vs. Control male	-0.09357	173	-0.5	0.62	0.96
		Control female vs. MPH female	-0.1713	171	-0.93	0.36	0.79
		Control female vs. MPH male	*0.4198*	*167*	*2.32*	*0.021*	*0.09*
		Control male vs. MPH female	-0.0777	168	-0.42	0.67	0.98
		**Control male vs. MPH male**	**0.5134**	**170**	**2.79**	**0.006**	**0.028**
		**MPH female vs. MPH male**	**0.5911**	**172**	**3.19**	**0.002**	**0.009**

Note: Bolded text indicates that a comparison between two groups is statistically significant at $P < 0.05$ after correcting for multiple comparisons; italicized text indicates that a comparison between two groups is less than $P < 0.1$ (but greater than $P = 0.05$) after correcting for multiple comparisons.

Table S4. Final results of the mixed model analyses of the open field tests for the G_2-G_4 cohorts with both G_1 female and G_1 male treatment as fixed effects. See a) for main effects and b) for random effects.

a) Main effects

Generation	Response	Original model	Final model	Estimate	DF_{num}	DF_{den}	*F* value	*P* value
G_2	CA1	G_1 female treatment * G_1 male treatment * sex * age, brood size, date tested, days until isolation, handling, time	Intercept	-0.5001		30.4		0.001
			G_1 female treatment	0.07901	1	26.8	0.37	0.55
			G_1 male treatment	-0.00664	1	32.8	0.001	0.96
			Age	-0.00348	1	270	5.42	0.021
			**G_1 male treatment * Age**	**-0.003**	**1**	**222**	**4.83**	**0.029**
			Sex	0.3371	1	276	7.15	0.008
			Sex * Age	0.00594	1	276	14.2	0.0002
			Time	0.0014	1	272	2.89	0.091
	CA2	G_1 female treatment * G_1 male treatment * sex * age, brood size, date tested, days until isolation, handling, time	Intercept	-0.1424		55.7		0.32
			G_1 female treatment	0.08173	1	54.9	0.37	0.54
			**G_1 male treatment**	**0.2747**	**1**	**62.1**	**4.28**	**0.04**
			Sex	0.1287	1	276	0.93	0.34
			Age	0.00086	1	107	0.54	0.46
			Sex * Age	-0.00414	1	274	5.99	0.02
			Date	0.00316	1	96.2	4.44	0.038
G_3	CA1	G_1 female treatment * G_1 male treatment * sex * age, brood size, date tested, days until isolation, handling, time	Intercept	-0.3479		132		0.091
			G_1 female treatment	-0.05487	1	43.8	0.13	0.72
			G_1 male treatment	0.1048	1	51.1	0.49	0.49
			Sex	0.03432	1	211	0.06	0.8
			Handling	0.07939	1	210	10.47	0.001
	CA2	G_1 female treatment * G_1 male treatment * sex * age, brood size, date tested, days until isolation, handling, time	Intercept	-0.6603		69.8		0.001
			G_1 female treatment	0.03094	1	50.7	0.17	0.86
			G_1 male treatment	0.1174	1	68.4	0.44	0.51
			Sex	0.7079	1	192	16.17	< 0.0001
			Age	-0.0055	1	194	7.34	0.002
			Sex * Age	0.0047	1	183	4.67	0.032

Table S4 continued

a) *Main effects (continued)*

Generation	Response	Original model	Final model	Estimate	DF_{num}	DF_{den}	*F* value	*P* value
G_4	CA1	G_1 female treatment * G_1 male treatment * sex * age, brood size, date tested, days until isolation, handling, time	Intercept	-0.4775		40.8		0.027
			G_1 female treatment	0.1473	1	27.5	0.1	0.75
			G_1 male treatment	0.1313	1	35.6	0.15	0.71
			Sex	0.3829	1	157	3.53	0.06
			G_1 male treatment * Sex	-0.1163	1	149	0.14	0.71
			Age	-0.00418	1	71.8	0.06	0.81
			G_1 male treatment * Age	0.0102	1	106	0.13	0.72
			Sex * Age	0.00803	1	156	0.02	0.88
			**G_1 male treatment * Sex * Age**	**-0.01727**	**1**	**154**	**4.69**	**0.03**
			Date	-0.00554	1	95	3.35	0.07
	CA2	G_1 female treatment * G_1 male treatment * sex * age, brood size, date tested, days until isolation, handling, time	Intercept	-0.3338		40.5		0.04
			G_1 female treatment	0.2779	1	33.6	1.69	0.1
			**G_1 male treatment**	**0.354**	**1**	**49.7**	**5.43**	**0.02**
			Sex	0.08067	1	163	0.31	0.58

Table S4 continued

b) *Random effects*

Generation	Response	Final model	Covariance Parameter	Estimate	SE	*Z* value	*P* value
G_2	CA1	G_1 female treatment, G_1 male treatment* Age, Sex*Age, Time	Pedigree	-0.05605	0.03882	-1.44	0.15
			Brood	0.1113	0.06813	1.63	0.05
			Residual	0.8877	0.09761	9.09	< 0.0001
	CA2	G_1 female treatment, G_1 male treatment, Sex*Age, Date	Pedigree	0.00807	0.06546	0.12	0.9
			Brood	0.05026	0.05899	0.85	0.2
			Residual	0.9155	0.09563	9.57	< 0.0001
G_3	CA1	G_1 female treatment, G_1 male treatment, Sex, Handling	Pedigree	-0.00953	0.05248	-0.18	0.86
			Brood	0.07712	0.07093	1.09	0.14
			Residual	0.8676	0.1033	8.4	< 0.0001
	CA2	G_1 female treatment, G_1 male treatment, Sex*Age	Pedigree	-0.0195	0.05835	-0.33	0.74
			Brood	0.05938	0.0578	1.03	0.15
			Residual	0.9511	0.0956	9.95	< 0.0001
G_4	CA1	G_1 female treatment, G_1 male treatment*Sex*Age, Date	Pedigree	0.1369	0.1937	0.71	0.48
			Brood	0.04501	0.1157	0.39	0.35
			Residual	0.7576	0.1451	5.22	< 0.0001
	CA2	G_1 female treatment, G_1 male treatment, Sex	Pedigree	0.00216	0.06873	0.03	0.97
			Brood	0.02632	0.06438	0.41	0.34
			Residual	0.8321	0.1096	7.59	< 0.0001

Table S5. Significance of slopes for final models for the analyses of CA1 for the G_2 and G_4 cohorts with significant interactions between G_1 male treatment and age (estimates for the final models are provided in Table 1).

Generation	Response	Group	*df*	*t* value	*P* value
G_2	CA1	Control Female	72.1	-0.47	0.64
		MPH Female	67.3	2.73	0.008
		Control Male	21.1	-3.37	0.003
		MPH male	25.3	-1.95	0.06
G_4	CA1	Control Female	30.6	-1.47	0.15
		MPH Female	30.5	2.49	0.02
		Control Male	42.5	0.08	0.93
		MPH male	25.6	-1.81	0.08

Table S6. Summary statistics for the age at testing, for each generation and sex separately.

Sex	Generation	N	Mean	Standard Deviation	Min	Q1	Median	Q3	Max
Female	G_1	95	132.1	42.62	69	98	132	170	227
	G_2	179	278.9	92.83	141	202	267	366	458
	G_3	115	383.2	70.57	136	342	394	434	494
	G_4	77	271	46.34	155	242	269	308	377
Male	G_1	95	127.71	39.95	69	96	117	156	213
	G_2	108	236.5	70.96	128	176	231	301	428
	G_3	102	292.4	62.38	152	236	300	332	443
	G_4	91	251.5	39.39	168	226	248	278	357

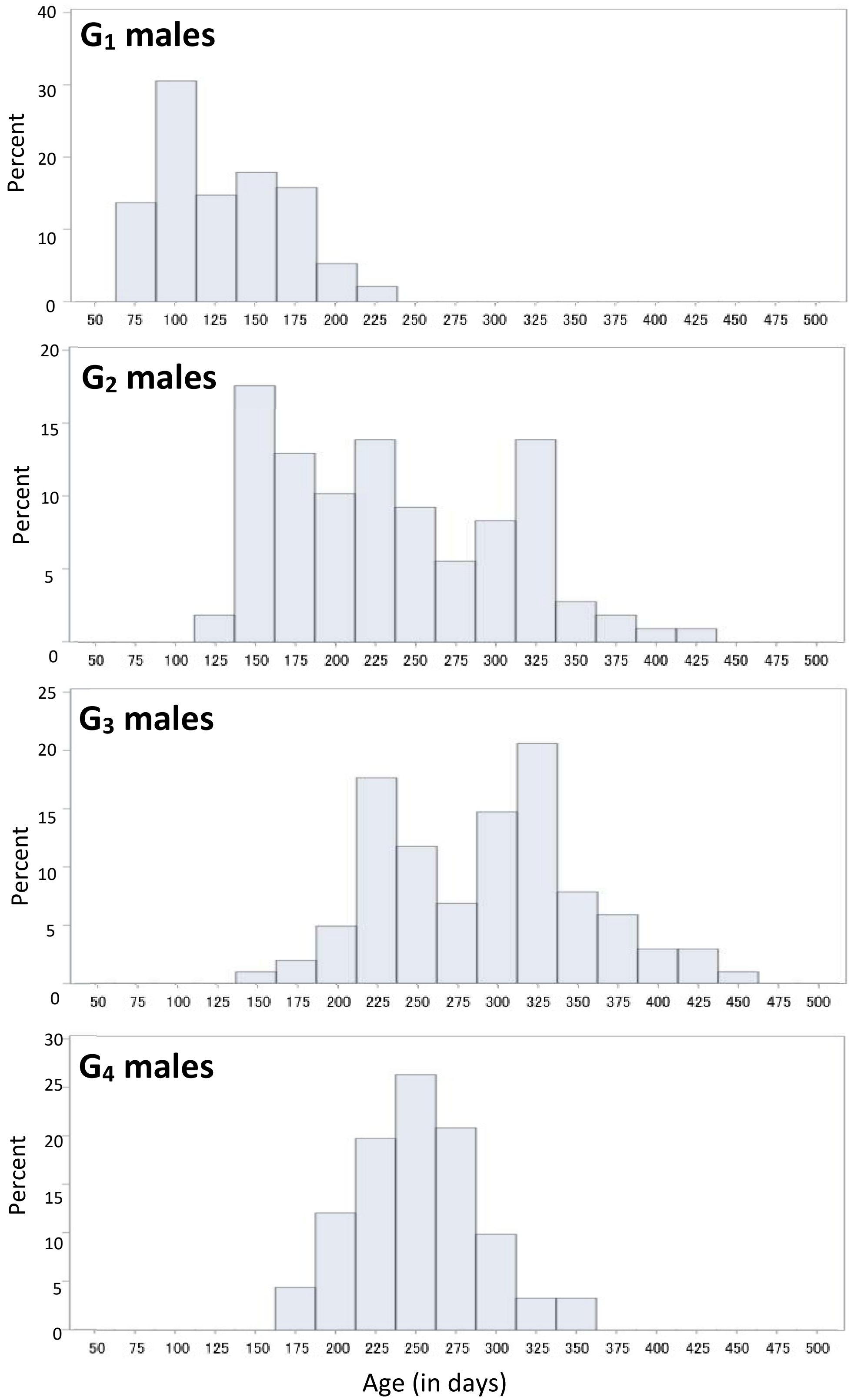

Figure S2. Histograms showing the age at testing in the open field test for males from each generation (G_1 – G_4).

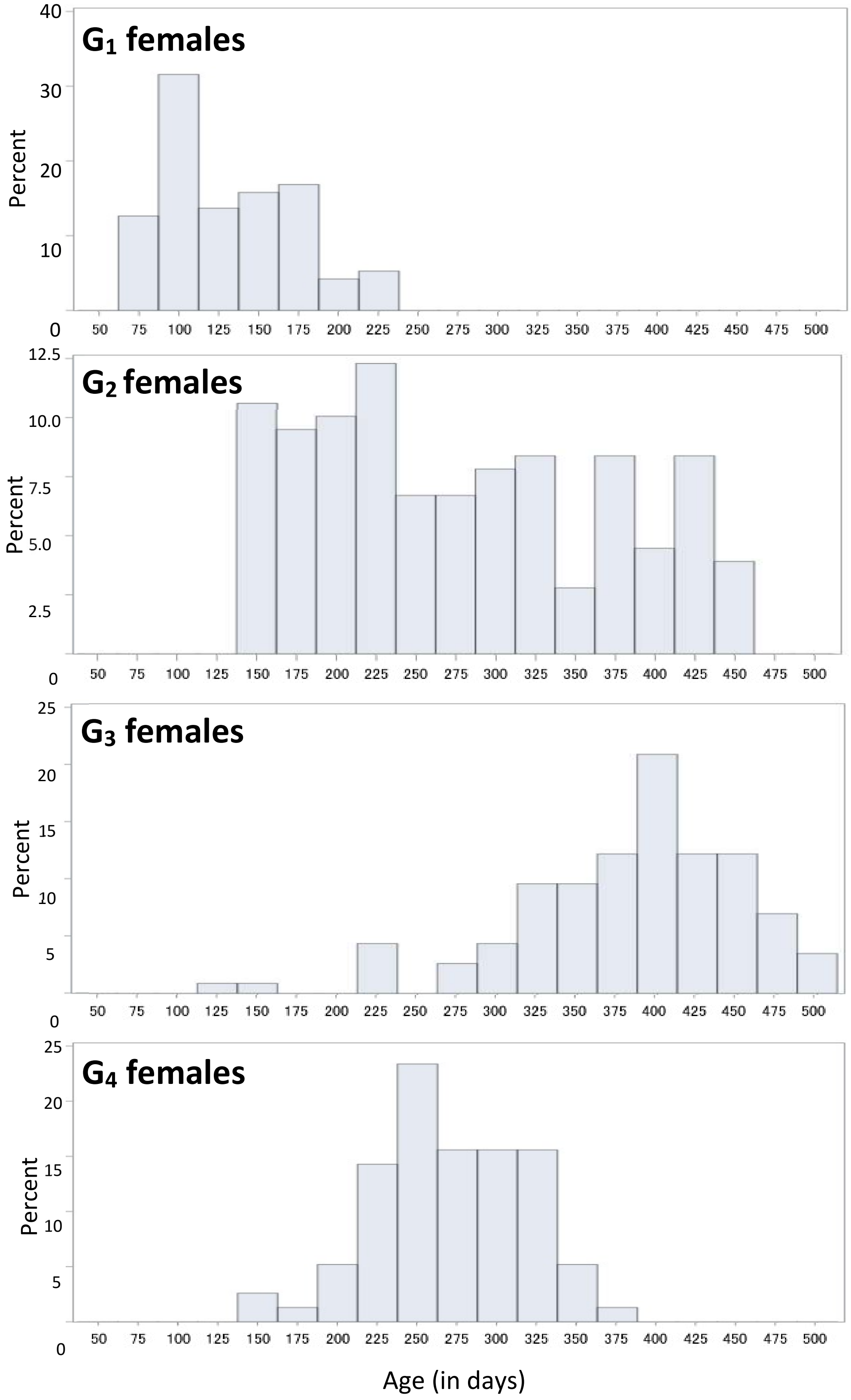

Figure S3. Histograms showing the age at testing in the open field test for females from each generation ($G_1 – G_4$).

Table S7. Final results of mixed model analyses of the open field test data with generation as a fixed effect for G_2 and G_4 'Control' males. See a) for main effects and b) for random effects.

a) Main effects

Group	Response	Original model	Final model	Estimate	DF_{num}	DF_{den}	*F* value	*P* value
Control Males	CA1	Generation * age, brood size, date tested, days until isolation, handling, time	Intercept	-0.9411		16.2		0.028
			Generation	-0.04965	1	6.85	0.02	0.9
			Handling	0.1238	1	83.9	6.32	0.014
	CA2	Generation * age, brood size, date tested, days until isolation, handling, time	Intercept	0.1755		6.56		0.49
			Generation	-0.1624	1	8.23	0.23	0.64
			Date	0.0043	1	88.3	4.6	0.035

b) Random effects

Group	Response	Final model	Covariance Parameter	Estimate	SE	*Z* value	*P* value
Control Males	CA1	Generation, handling	Pedigree	0.2871	0.3126	0.92	0.36
			Brood	0.04284	0.1929	0.22	0.41
			Residual	1.0279	0.2777	3.7	0.0001
	CA2	Generation, date	Pedigree	0.1947	0.2126	0.92	0.36
			Brood	0.2433	0.1434	1.7	0.045
			Residual	0.6084	0.183	3.33	0.0004

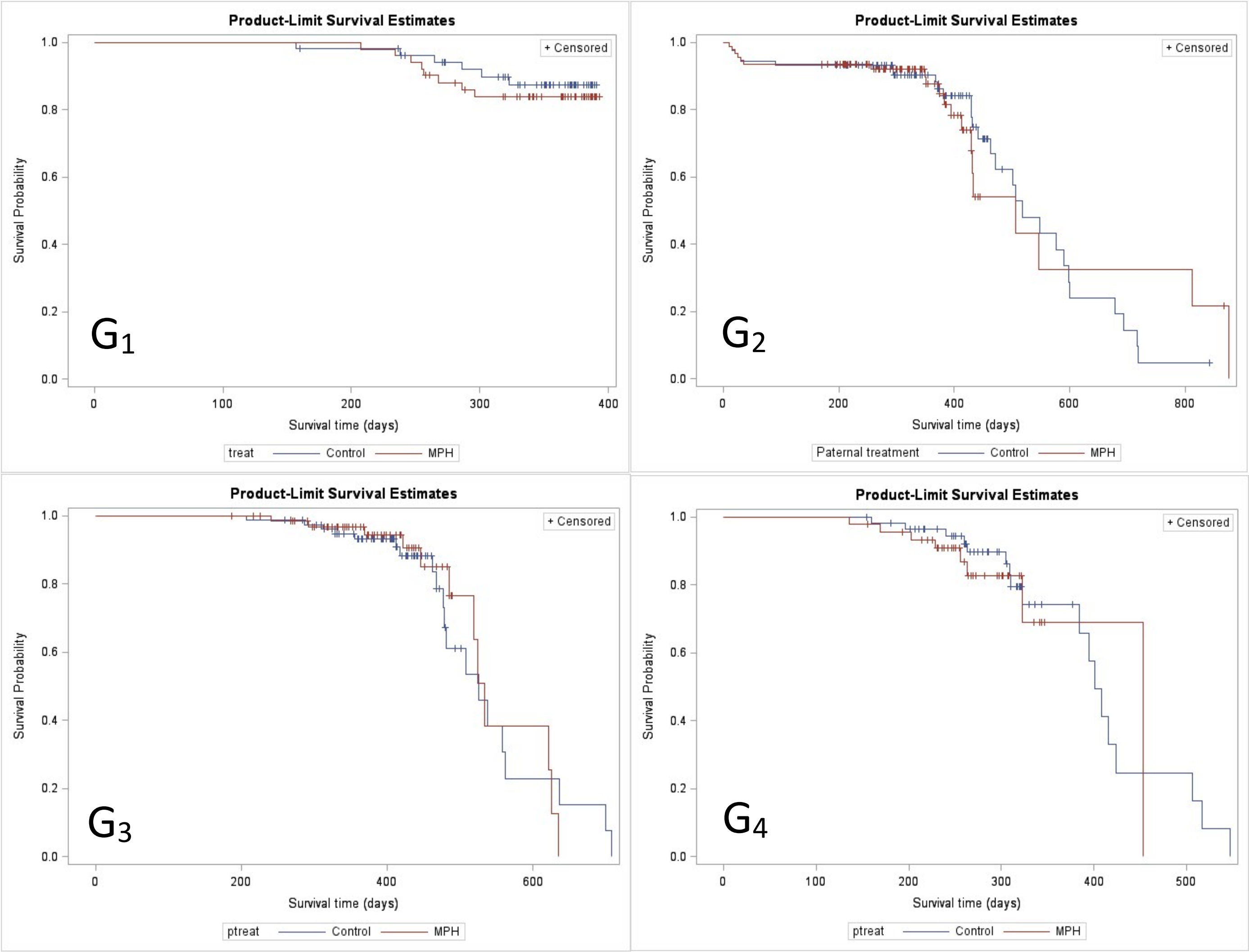

Figure S4. To determine if MPH affected female survival, Kaplan-Meier survival analyses were run for each generation (G_1 to G_4). Survival estimates did not significantly differ between Control and MPH treated females for any cohort. Censored individuals are females who were sacrificed for dissection.

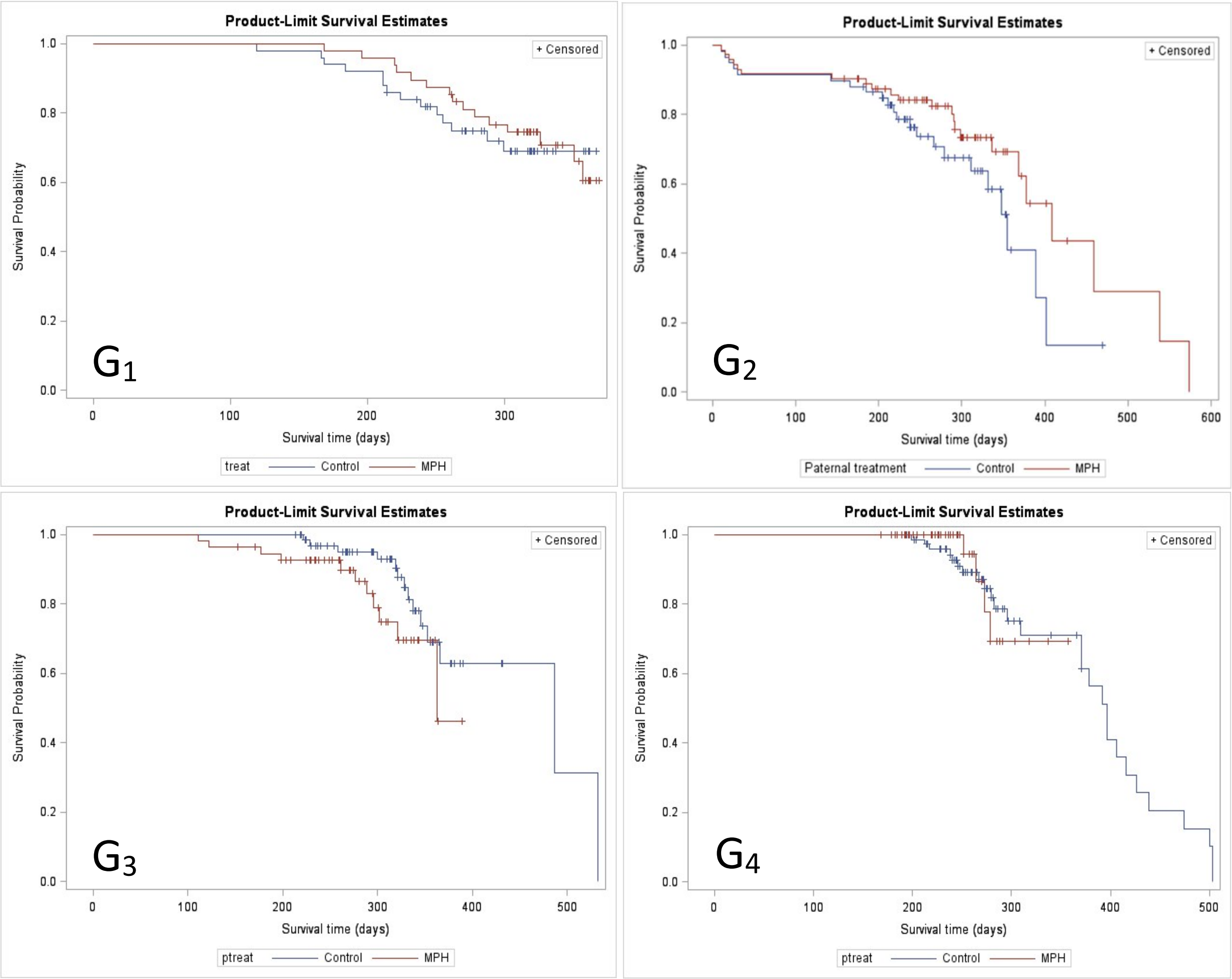

Figure S5. To determine if MPH affected male survival, Kaplan-Meier survival analyses were run for each generation (G_1 to G_4). Survival estimates did not significantly differ between Control and MPH treated males for any cohort. Censored individuals are males who were sacrificed for dissection.

Table S8. Survival and censorship data for Kaplan-Meier survival analyses of G_1-G_4 females and males. Fish were classified as censored if they were sacrificed for dissection.

**Sex**	**Generation**	**Treatment**	**Total n**	**Died**	**Censored**	**χ^2 stat**	***df***	***P***
Female	G_1	Control	54	6	48	0.381	1	0.54
		MPH	51	8	43			
	G_2	Control	90	29	61	0.009	1	0.92
		MPH	93	20	73			
	G_3	Control	81	20	61	0.01	1	0.92
		MPH	69	12	57			
	G_4	Control	57	18	39	0.275	1	0.6
		MPH	46	8	38			
Male	G_1	Control	50	14	36	0.111	1	0.74
		MPH	48	15	33			
	G_2	Control	59	22	37	2.62	1	0.11
		MPH	72	23	49			
	G_3	Control	69	14	55	2.7	1	0.1
		MPH	56	11	45			
	G_4	Control	75	27	48	0.003	1	0.95
		MPH	48	4	44			

Table S9. Sires and dams from the G_1-G_3 cohorts who produced offspring ("Yes") or did not produce offspring ("No"). For the G_1 cohort, sires and dams were administered MPH or were Controls. For the G_2 and G_3 cohorts, 'Control' or 'MPH treated' refers to the treatment group of their sire (G_2 cohort) or their grandsire (G_3 cohort).

**G_1 Sires**		
	Control	MPH treated
Yes	21	20
No	21	17
	P = 0.822	

**G_1 Dams**		
	Control	MPH treated
Yes	27	22
No	15	24
	P = 0.138	

**G_2 Sires**		
	Control	MPH treated
Yes	18	20
No	3	4
	P = 1	

**G_2 Dams**		
	Control	MPH treated
Yes	21	21
No	3	5
	P = 0.704	

**G_3 Sires**		
	Control	MPH treated
Yes	14	13
No	10	12
	P = 0.776	

**G_3 Dams**		
	Control	MPH treated
Yes	16	14
No	6	7
	P = 0.747	

Table S10. The number of offspring that were produced by sires and dams from the G_1, G_2 and G_3 cohorts. For the G_1 cohort, sires and dams were administered MPH or were Controls.

	Mann-Whitney *U*	n	*P* value
**G_1 Sires**	218.5	43	0.77
**G_2 Sires**	159.5	38	0.55
**G_3 Sires**	95.5	29	0.73

	Mann-Whitney *U*	n	*P* value
**G_1 Dams**	265.5	48	0.65
**G_2 Dams**	196.5	42	0.55
**G_3 Dams**	125.5	32	1

Table S11. The numbers of Control and MPH treated sires and dams from the G_1, G_2 and G_3 cohorts that produced either one brood or more than one brood (2+). For the G_1 cohort, sires and dams were administered MPH or were Controls. For the G_2 and G_3 cohorts, 'Control' or 'MPH treated' refers to the treatment group of their sire (G_2 cohort) or their grandsire (G_3 cohort).

**G_1 Sires**			
Broods	Control	MPH treated	*P* value
1	8	6	0.79
2+	14	16	0.86
**G_2 Sires**			
Broods	Control	MPH treated	*P* value
1	6	6	1
2+	13	13	1
**G_3 Sires**			
Broods	Control	MPH treated	*P* value
1	6	4	0.75
2+	11	10	1

**G_1 Dams**			
Broods	Control	MPH treated	*P* value
1	11	5	0.22
2+	14	17	0.72
**G_2 Dams**			
Broods	Control	MPH treated	*P* value
1	7	9	0.8
2+	14	12	0.85
**G_3 Dams**			
Broods	Control	MPH treated	*P* value
1	7	4	0.55
2+	12	10	0.83

Table S12. Analyses of the sex ratio of offspring produced by fish in the G_0-G_3 cohorts. For the G_2-G_4 cohorts, 'Control' or 'MPH' refers to the treatment group of their sire (G_2 cohort), grandsire (G_3 cohort), or great-grandsire (G_4 cohort).

**G_1 sex ratio**				
Group	Female	Male	Proportion Male	*P* value
-	138	128	0.481	0.581

**G_2 sex ratio**			
Group	Female	Male	Proportion Male
Control	96	88	0.478
MPH	98	91	0.481
	P =1		
**G_3 sex ratio**			
Group	Female	Male	Proportion Male
Control	91	83	0.477
MPH	78	60	0.435
	P = 0.49		
**G_4 sex ratio**			
Group	Female	Male	Proportion Male
Control	67	85	0.559
MPH	48	50	0.51
	P = 0.52		

Table S13. Final results of the sex ratio analyses of offspring produced by mated pairs in the G_1-G_4 cohorts that survived to maturity. See a) for main effects and b) for random effects.

a) Main effects

Generation	Final model	Estimate	DF_{num}	DF_{den}	*t* value	*P* value	Dispersion*	n Pairs	n Males	n Total
G_1	Intercept	-0.07522		1	-0.61	0.65	0.87	18	128	266

Generation	Final model	Estimate	DF_{num}	DF_{den}	*F* value	*P* value	Dispersion*	n Pairs	n Males	n Total
G_2	Intercept	-0.07411		1		0.7	1.07	49	179	373
	G_1 male treatment	-0.0129	1	1	0.001	0.96				
G_3	Intercept	-0.2502		26.56		0.18	0.97	42	143	312
	G_1 male treatment	0.1574	1	36.05	0.41	0.52				
G_4	Intercept	-0.1355		6.77		0.77	0.9	33	135	250
	G_1 male treatment	0.3482	1	10.19	0.27	0.61				

*Note: Dispersion values of ~1 indicate the data is following a binomial distribution. "n Pairs" indicates the number of mating pairs (from the previous generation), "n Males" indicates the number of male offspring, and "n Total" indicates the total number of offspring.

Supplemental Table S13 (continued)

b) Random effects

Generation	Final model	Covariance Parameter	Estimate	SE
G_1	Intercept	Line	0	.
G_2	G_1 male treatment	Maternal line	0	.
		Paternal line	0	.
		Maternal * Paternal line	0	.
G_3	G_1 male treatment	Maternal Grandmother	0	.
		Maternal Grandfather	0	.
		Paternal Grandmother	0.01481	0.0758
		Paternal Grandfather	0	.
		Maternal Grandmother*Maternal Grandfather	0	.
		Paternal Grandmother*Paternal Grandfather	0	.
		Maternal Grandmother*Maternal Grandfather* Paternal Grandmother*Paternal Grandfather	0	.
G_4	G_1 male treatment	Mother of Maternal Grandmother (MMGM)	0.06595	0.1989
		Father of Maternal Grandmother (FMGM)	0	.
		Mother of Maternal Grandfather (MMGF)	0	.
		Father of Maternal Grandfather (FMGF)	0	.
		Mother of Paternal Grandmother (MPGM)	0	.
		Father of Paternal Grandmother (FPGM)	0.02585	0.1589
		Mother of Paternal Grandfather (MPGF)	0.2909	0.7341
		Father of Paternal Grandfather (FPGF)	0	.
		MMGM * FMGM	0	.
		MMGF * FMGF	0	.
		MPGM * FPGM	0	.
		MPGF * FPGF	0.4127	0.7378
		MMGM * FMGM * MMGF * FMGF	0	.
		MPGM * FPGM * MPGF * FPGF	0	.
		MMGM * FMGM * MMGF * FMGF * MPGM * FPGM * MPGF * FPGF	0	.

Table S14. Dopamine concentrations (ng/mL) of the standards that were used to produce the standard curve for each run. The known concentration of the standard is indicated in brackets; differences between the known concentration and the concentration calculated for each run are due to measurement and experimental error.

Run	Generation	Date run	Dopamine Concentration (ng/mL) of Standards					
			Std A **(0)**	Std B **(0.5)**	Std C **(1.5)**	Std D **(5)**	Std E **(20)**	Std F **(80)**
1	G2	May 11 2014	n/a	0.507	1.435	5.282	19.356	80.693
2	G2 & G3	Apr 26 2015	0.000	0.276	2.028	5.239	18.666	81.372
3	G3	May 3 2015	< 0.0001	0.520	1.354	5.564	18.873	81.228
4	G3 & G4	May 10 2015	< 0.0001	0.637	1.190	5.160	20.572	79.014
5	G4	Sept 24 2017	0.017	0.613	1.133	5.683	18.345	> 84.000
6	G4	Sept 26 2017	< 0.0001	0.599	1.266	4.910	21.982	76.138
7	G4	Sept 28 2017	< 0.0001	0.518	1.516	4.787	20.620	79.157
8	G4	Oct 1 2017	< 0.0001	0.515	1.559	4.493	21.702	77.848

Table S15. Final results of mixed model analyses of whole brain dopamine concentration for all generations for which data were available: females from the G_2 and males and females from G_3 and G_4. See a) for main effects and b) for random effects.

a) Main effects

Generation	Sex	Original model	Final model	Estimate	DF_{num}	DF_{den}	*F* value	*P* value
G_2	Female	G_1 male treatment * age * brain weight	Intercept	3.5158		1		0.2
			G_1 male treatment	-0.1838	1	12.6	0.44	0.52
			Age	0.005	1	63.4	3.87	0.053
G_3	Female	G_1 male treatment * age * brain weight	Intercept	1.9255		1		0.13
			G_1 male treatment	-0.01707	1	61.5	0.03	0.87
			Brain weight	180.24	1	84.4	12.86	0.0006
	Male	G_1 male treatment * age * brain weight	Intercept	1.2858		2.19		0.064
			G_1 male treatment	-0.00898	1	53.4	0.01	0.93
G_4	Female	G_1 male treatment * age * brain weight	Intercept	1.6685		3.65		0.01
			G_1 male treatment	0.03633	1	41.2	0.04	0.85
			Brain weight	205.43	1	34.1	3.87	0.057
	Male	G_1 male treatment * age * brain weight	Intercept	1.1252		4.14		0.036
			G_1 male treatment	-0.01494	1	13.3	0.03	0.87
			Brain weight	64.4495	1	49.7	1.29	0.26
			Age	0.00087	1	50.8	-0.73	0.4
			Brain weight * Age	3.1175	1	47.7	4.36	0.04

Table S15 continued.

b) Random effects

Generation	Sex	Final model	Covariance Parameter	Estimate	SE	*Z* value	*P* value
G_2	Female	G_1 male treatment, age	Pedigree	-0.08948	0.04901	-1.83	0.068
			Run	2.4914	3.6315	0.69	0.25
			Residual	1.3248	0.2417	5.48	< 0.0001
G_3	Female	G_1 male treatment, brain weight	Pedigree	-0.01599	0.00719	-2.23	0.03
			Run	0.3301	0.4768	0.69	0.24
			Residual	0.224	0.0364	6.15	< 0.0001
	Male	G_1 male treatment	Pedigree	-0.00091	0.01134	-0.08	0.94
			Run	0.3701	0.3863	0.96	0.17
			Residual	0.1375	0.02813	4.89	< 0.0001
G_4	Female	G_1 male treatment, brain weight	Pedigree	-0.03	0.02326	-1.29	0.2
			Run	0.5744	0.4922	1.17	0.12
			Residual	0.4158	0.09441	4.4	< 0.0001
	Male	G_1 male treatment, brain weight*age	Mother of Maternal Grandmother (MMGM)	0	.	.	.
			Father of Maternal Grandmother (FMGM)	0	.	.	.
			Mother of Maternal Grandfather (MMGF)	0.01002	0.01265	0.79	0.21
			Father of Maternal Grandfather (FMGF)	0	.	.	.
			Mother of Paternal Grandmother (MPGM)	0	.	.	.
			Father of Paternal Grandmother (FPGM)	0	.	.	.
			Mother of Paternal Grandfather (MPGF)	0	.	.	.
			Father of Paternal Grandfather (FPGF)	0	.	.	.
			MMGM * FMGM	0	.	.	.
			MMGF * FMGF	0	.	.	.
			MPGM * FPGM	0	.	.	.
			MPGF * FPGF	0	.	.	.
			MMGM * FMGM * MMGF * FMGF	0	.	.	.
			MPGM * FPGM * MPGF * FPGF	0	.	.	.
			MMGM * FMGM * MMGF * FMGF * MPGM * FPGM * MPGF * FPGF	0	.	.	.
			Run	0.6534	0.4667	1.4	0.08
			Residual	0.04649	0.01053	4.41	< 0.0001

Supplemental Table S16. Final results of mixed model analyses of open field trials for inner/total squares traversed for the G_1-G_4 cohorts.

a) *Main effects*

Generation	Original model	Final model	Estimate	DF_{num}	DF_{den}	*F* value	*P* value*
G_1	Treatment * sex * age, brood size, date tested, days until isolation, handling, time	Intercept	0.4185		53		<0.0001
		Treatment	0.05866	1	164	1.5	0.22
		sex	-0.00116	1	169	5.14	0.025
		**Treatment*sex**	**-0.0759**	**1**	**175**	**4.53**	**0.034**
G_2	G_1 male treatment * sex * age, brood size, date tested, days until isolation, handling, time	Intercept	0.4167		124		< 0.0001
		G_1 male treatment	-0.03603	1	64	0.4	0.53
		Sex	-0.02274	1	275	0.04	0.84
		G_1 male treatment * Sex	0.05144	1	272	3.31	0.07
		Age	-0.00041	1	277	11.59	0.0008
		**G_1 male treatment * Age**	**-0.00034**	**1**	**272**	**4.44**	**0.036**
		Sex * Age	0.00056	1	278	9.63	0.002
G_3	G_1 male treatment * sex * age, brood size, date tested, days until isolation, handling, time	Intercept	0.551		86		< 0.0001
		G_1 male treatment	-0.02158	1	61.2	1.08	0.3
		Sex	-0.09535	1	209	18.73	< 0.0001
		Age	0.0005	1	188	2.4	0.12
		Sex * Age	-0.00055	1	206	3.97	0.048
		Time	-0.00046	1	205	3.64	0.058
G_4	G_1 male treatment * sex * age, brood size, date tested, days until isolation, handling, time	Intercept	0.4681		44.6		< 0.0001
		G_1 male treatment	0.02102	1	35.9	0.001	0.95
		Sex	-0.0014	1	157	0.84	0.36
		G_1 male treatment * Sex	-0.03886	1	148	0.76	0.38
		Age	-0.00077	1	112	0.96	0.33
		G_1 male treatment * Age	0.00114	1	115	0.001	0.99
		Sex * Age	0.00098	1	158	0.1	0.76
		**G_1 male treatment * Sex * Age**	**-0.00231**	**1**	**158**	**4.2**	**0.042**

b) *Random effects*

Generation	Final model	Covariance Parameter	Estimate	SE	*Z* value	*P* value
G_1	Treatment * Sex	Line	0.00224	0.00239	0.94	0.17
		Brood	0.002	0.00249	0.8	0.21
		Residual	0.01295	0.00147	8.83	< 0.0001
G_2	G_1 male treatment * age, G_1 male treatment * sex, sex * age	Pedigree	-0.00062	0.00051	-1.21	0.23
		Brood	0.00183	0.00091	2.01	0.022
		Residual	0.01148	0.00116	9.94	< 0.0001
G_3	G_1 male treatment, sex * age, time	Pedigree	0.00043	0.0012	0.36	0.72
		Brood	0.00112	0.0011	1.04	0.15
		Residual	0.0151	0.00186	8.1	< 0.0001
G_4	G_1 male treatment * sex * age	Pedigree	0.00226	0.00277	0.81	0.42
		Brood	0.00175	0.0024	0.73	0.23
		Residual	0.01554	0.00264	5.9	< 0.0001

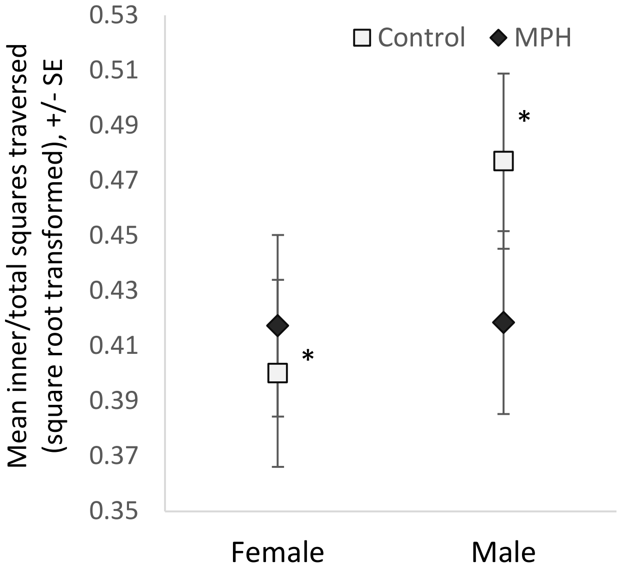

Figure S6. Inner/total squares traversed by first generation (G_1) male and female guppies in the open field test. Control males swam through significantly more inner squares (relative to total squares) than Control females, and there was a non-significant trend for them to swim through more inner squares than both MPH treated groups. Symbols represent least-square means for each response variable, +/- one standard error. Asterisks indicate points that are significantly different from one another at $P < 0.05$.

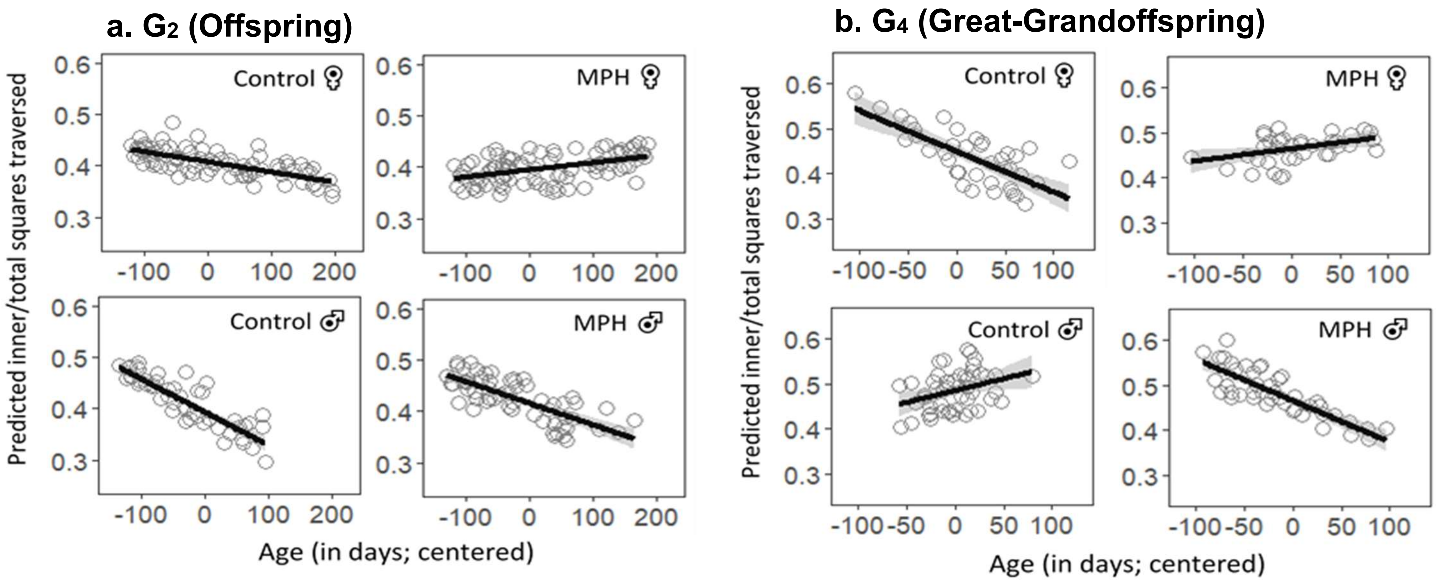

Figure S7. Associations between age and inner/total squares traversed (square root transformed) in the open field test for offspring (G_2; panel a) and great-grandoffspring (G_4; panel b). Results were qualitatively the same as for offspring and great-grandoffspring CA1 scores (see main text). Lines were fit to the predicted values (which incorporate model estimates and random effects) using 'lm' in R and shading corresponds to the 95% confidence intervals.

Table S17. Final results of mixed model analyses of activity levels (total squares traversed) in the open field test for all cohorts where there were significant behavioural effects of G_1 male treatment. See a) for main effects and b) for random effects.

a) Main effects

Generation	Original model	Final model	Estimate	DF_{num}	DF_{den}	*F* value	*P* value
G_1	Treatment * sex * age, brood size, date tested, days until isolation, handling, time	Intercept	230.13		15		< 0.0001
		Treatment	-11.7482	1	148	2.33	0.13
		Sex	-38.7871	1	167	23.65	< 0.0001
		Date	-0.2785	1	165	12.97	0.0004
G_2	G_1 male treatment * sex * age, brood size, date tested, days until isolation, handling, time	Intercept	224.65		178		0.0004
		G_1 male treatment	-7.5778	1	65.4	0.96	0.33
		Age	-0.06809	1	262	0.001	0.95
		G_1 male treatment * Age	0.1314	1	255	2.78	0.097
		Sex	-29.2176	1	270	16.89	< 0.0001
		Handling	-3.3276	1	256	7.78	0.006
G_4	G_1 male treatment * sex * age, brood size, date tested, days until isolation, handling, time	Intercept	197.49		29		< 0.0001
		G_1 male treatment	-5.5231	1	36.7	0.33	0.57
		Sex	-33.0651	1	154	13.73	0.0003
		Date	0.3745	1	134	7.83	0.006

b) Random effects

Generation	Final model	Covariance Parameter	Estimate	SE	*Z* value	*P* value
G_1	Treatment * Sex, Date	Line	307.04	207.85	1.48	0.07
		Brood	0	.	.	.
		Residual	2735.53	322.84	8.47	< 0.0001
G_2	G_1 male treatment * Age, Sex, handling	Pedigree	-143.76	139.64	-1.03	0.3
		Brood	353.22	226.19	1.56	0.06
		Residual	2969.02	292.55	10.15	< 0.0001
G_4	G_1 male treatment, Sex, date	Pedigree	896.89	574.51	1.56	0.12
		Brood	84.18	251.54	0.33	0.37
		Residual	2089.68	417.66	5	< 0.0001

Table S18. Correlation between age and body size (standard length) for all generational cohorts for each sex separately.

Females- Correlation between age and standard length

	Pearson's correlation				Spearman's rank correlation		
**Generation**	***t* statistic**	***df***	**Correlation**	***P* value**	***S* statistic**	**rho (correlation)**	***P* value**
G_1	-0.54	82	-0.06	0.59	102710	-0.04	0.72
G_2	0.53	105	0.05	0.6	200710	0.02	0.86
**G_3**	**-2.95**	**107**	**-0.27**	**0.004**	**266010**	**-0.23**	**0.01**
G_4	1.03	73	0.12	0.31	56527	0.2	0.09

Males- Correlation between age and standard length

	Pearson's correlation				Spearman's rank correlation		
**Generation**	***t* statistic**	***df***	**Correlation**	***P* value**	***S* statistic**	**rho (correlation)**	***P* value**
G_1	1.01	68	0.12	0.31	50354	0.12	0.33
G_2	-1.08	85	-0.12	0.28	117580	-0.07	0.51
G_3	-1.45	85	-0.16	0.15	115710	-0.05	0.62
G_4	-0.74	87	-0.08	0.46	118900	-0.01	0.91

Table S19. Final results of mixed model analyses of the open field tests for the subset of individuals with body size (standard length) measurements for the G_1-G_4 cohorts. See a) for main effects and b) for random effects.

a) *Main effects*

Generation	Sex	Response	Original model	Final model	Estimate	DF_{num}	DF_{den}	*F* value	*P* value
G_1	Females	CA1	Treatment * Age * Standard Length, Time, Date tested, Days until isolation, Brood size, Handling	Intercept	0.1037		8.3		0.620
				Treatment	-0.2456	1	69.1	1.05	0.31
				Standard length	0.00751	1	78.2	0.02	0.9
		CA2	Treatment * Age * Standard Length, Time, Date tested, Days until isolation, Brood size, Handling	Intercept	0.926		71.2		0.01
				Treatment	0.07635	1	60.8	0.12	0.73
				Standard length	0.01631	1	71.5	0.08	0.78
				Handling	-0.06983	1	75.4	3.19	0.078
	Males	CA1	Treatment * Age * Standard Length, Time, Date tested, Days until isolation, Brood size, Handling	Intercept	-0.6721		25.9		0.002
				Treatment	0.6456	1	47	8.07	0.007
				Standard length	0.7821	1	50.3	0.65	0.42
		CA2	Treatment * Age * Standard Length, Time, Date tested, Days until isolation, Brood size, Handling	Intercept	0.1601		8.88		0.45
				Treatment	-0.1512	1	58.2	0.43	0.51
				Age	0.00499	1	56.4	3.09	0.08
				Standard length	-0.9738	1	53.2	0.93	0.34

Table S19 continued

a) *Main effects (continued)*

Generation	Sex	Response	Original model	Final model	Estimate	DF_{num}	DF_{den}	*F* value	*P* value
G_2	Females	CA1	G_1 male treatment * Age * Standard Length, Time, Date tested, Days until isolation, Brood size, Handling	Intercept	0.1718		19.6		0.34
				G_1 male treatment	0.01549	1	31.7	0.001	0.94
				Age	-0.00371	1	86.7	5.73	0.02
				G_1 male treatment*Age	-0.0068	1	89.5	6.81	0.01
				Standard length	0.1461	1	90.1	0.06	0.81
				Date	0.00639	1	72.8	6.53	0.01
				Brood Size (1 to 2)	-0.7106	2	64.4	3.1	0.052
				Brood Size (3 to 6)	-0.5383				
		CA2	G_1 male treatment * Age * Standard Length, Time, Date tested, Days until isolation, Brood size, Handling	Intercept	0.699		65.5		0.01
				G_1 male treatment	0.3919	1	48.2	2.61	0.11
				Standard length	-0.3581	1	90	0.31	0.58
				handling	-0.1064	1	94.7	8.31	0.005
	Males	CA1	G_1 male treatment * Age * Standard Length, Time, Date tested, Days until isolation, Brood size, Handling	Intercept	-0.8514		53		0.0004
				G_1 male treatment	0.05185	1	81.9	0.07	0.79
				Age	-0.00471	1	79.8	12.13	0.0008
				Standard length	-0.235	1	81.5	0.05	0.82
				handling	0.104	1	81.8	10.61	0.002
		CA2	G_1 male treatment * Age * Standard Length, Time, Date tested, Days until isolation, Brood size, Handling	Intercept	-0.4638		54.8		0.066
				G_1 male treatment	-0.2183	1	79.9	1.19	0.28
				Age	-0.00189	1	64	0.63	0.43
				Standard length	1.5319	1	78	2.28	0.13
				**Age * Standard length**	**0.02829**	**1**	**77.1**	**4.13**	**0.046**
				Date	0.00687	1	63.7	8.23	0.006
				handling	0.08181	1	72.5	7.39	0.008

Table S19, Main effects (continued)

Generation	Sex	Response	Original model	Final model	Estimate	DF_{num}	DF_{den}	*F* value	*P* value
G_3	Females	CA1	G_1 male treatment * Age * Standard Length, Time, Date tested, Days until isolation, Brood size, Handling	Intercept	-0.1991		44.8		0.51
				G_1 male treatment	0.08031	1	32.4	0.12	0.74
				Standard length	-0.2682	1	96.7	0.32	0.57
				handling	0.06223	1	101	3.37	0.069
		CA2	G_1 male treatment * Age * Standard Length, Time, Date tested, Days until isolation, Brood size, Handling	Intercept	-0.01028		13.8		0.96
				G_1 male treatment	0.204	1	43.8	0.57	0.46
				Standard length	0.01921	1	94.3	0.001	0.97
	Males	CA1	G_1 male treatment * Age * Standard Length, Time, Date tested, Days until isolation, Brood size, Handling	Intercept	-0.6071		66.8		0.04
				G_1 male treatment	-0.1251	1	31.9	0.29	0.59
				Standard length	-0.266	1	81.7	1.13	0.29
				Days until isolation	0.00402	1	81	11.26	0.001
				handling	0.1363	1	79.1	7.75	0.007
		CA2	G_1 male treatment * Age * Standard Length, Time, Date tested, Days until isolation, Brood size, Handling	Intercept	-0.3776		11.9		0.037
				G_1 male treatment	0.07613	1	61.1	0.12	0.73
				Age	-0.00584	1	82.2	14.03	0.0003
				Standard length	0.06811	1	81.9	0.09	0.76
G_4	Females	CA1	G_1 male treatment * Age * Standard Length, Time, Date tested, Days until isolation, Brood size, Handling	Intercept	0.01504		3.14		0.94
				G_1 male treatment	-0.1393	1	14.3	0.27	0.61
				**Standard length**	**0.7072**	**1**	**62.2**	**6.65**	**0.012**
		CA2	G_1 male treatment * Age * Standard Length, Time, Date tested, Days until isolation, Brood size, Handling	Intercept	-0.02424		4.9		0.89
				G_1 male treatment	0.218	1	16.8	0.69	0.42
				Standard length	-0.2551	1	64.8	0.76	0.39
	Males	CA1	G_1 male treatment * Age * Standard Length, Time, Date tested, Days until isolation, Brood size, Handling	Intercept	-0.2344		10.1		0.36
				G_1 male treatment	-0.05996	1	43.5	0.05	0.83
				Standard length	0.7742	1	87	2.3	0.13
		CA2	G_1 male treatment * Age * Standard Length, Time, Date tested, Days until isolation, Brood size, Handling	Intercept	-0.3399		1		0.33
				G_1 male treatment	0.5635	1	9.69	6.03	0.035
				Standard length	0.131	1	18.4	0.06	0.81

Table S19, b) Random effects

Generation	Sex	Response	Final Model	Covariance Parameter	Estimate	SE	Z value	P value
G_1	Female	CA1	Treatment, Standard length	Line	0.06198	0.1111	0.56	0.29
				Brood	0	.	.	.
				Residual	0.9606	0.1835	5.23	< 0.0001
		CA2	Treatment, Standard length, Handling	Line	0.266	0.1559	1.71	0.04
				Brood	0	.	.	.
				Residual	0.6959	0.1459	4.77	< 0.0001
	Male	CA1	Treatment, Standard length	Line	0.1403	0.2798	0.5	0.31
				Brood	0.1109	0.3444	0.32	0.37
				Residual	0.4631	0.1344	3.45	0.0003
		CA2	Treatment, Age, Standard length	Line	0.04228	0.4215	0.1	0.46
				Brood	0.08931	0.4156	0.21	0.42
				Residual	0.535	0.1105	4.84	<0.0001
G_2	Female	CA1	G_1 male treatment*Age, Standard length, Date, Brood number	Pedigree	-0.0525	0.05285	-0.99	0.32
				Brood	0.1495	0.1328	1.13	0.13
				Residual	0.8946	0.1706	5.24	<0.0001
		CA2	G_1 male treatment, Standard length, Handling	Pedigree	0.02074	0.1159	0.18	0.86
				Brood	0.1133	0.1429	0.79	0.21
				Residual	1.0248	0.1819	5.63	<0.0001
	Male	CA1	G_1 male treatment, Standard length, Age, Handling	Pedigree	0.00187	0.0662	0.03	0.98
				Brood	0	.	.	.
				Residual	0.738	0.1297	5.69	<0.0001
		CA2	G_1 male treatment, Standard length*Age, Date, Handling	Maternal line	0	.	.	.
				Paternal line	0.2144	0.132	1.62	0.05
				Maternal*Paternal line	0	.	.	.
				Brood	0	.	.	.
				Residual	0.5806	0.1012	5.74	<0.0001
G_3	Female	CA1	G_1 male treatment, Standard length, Handling	Pedigree	-0.01333	0.1018	-0.13	0.9
				Brood	0.07784	0.1481	0.53	0.3
				Residual	1.015	0.1827	5.56	<0.0001
		CA2	G_1 male treatment, Standard length	Pedigree	0.1797	0.2349	0.77	0.44
				Brood	0.1343	0.1737	0.77	0.22
				Residual	1.177	0.2414	4.88	<0.0001
	Male	CA1	G_1 male treatment, Standard length, Days until isolated, Handling	Pedigree	-0.03987	0.05391	-0.74	0.46
				Brood	0	.	.	.
				Residual	0.7932	0.1392	5.7	<0.0001
		CA2	G_1 male treatment, Standard length, Age	Pedigree	0.01611	0.0888	0.18	0.86
				Brood	0.221	0.1057	2.09	0.02
				Residual	0.4434	0.1056	4.2	<0.0001

Table S19, b) Random effects (continued)

Generation	Sex	Response	Final Model	Covariance Parameter	Estimate	SE	Z value	P value
G_4	Female	CA1	G_1 male treatment, Standard length	Pedigree	-0.05375	0.05852	-0.92	0.36
				Brood	0.0451	0.1352	0.33	0.37
				Residual	0.8257	0.169	4.89	<0.0001
		CA2	G_1 male treatment, Standard length	Pedigree	-0.05252	0.05544	-0.95	0.34
				Brood	0.08177	0.1526	0.54	0.3
				Residual	0.8975	0.2023	4.44	<0.0001
	Male	CA1	G_1 male treatment, Standard length	Pedigree	0.2406	0.266	0.9	0.37
				Brood	0.2248	0.1711	1.31	0.09
				Residual	0.5932	0.1958	3.03	0.001
		CA2	G_1 male treatment, Standard length	Pedigree	-0.0827	0.03342	-2.47	0.01
				Brood	0.187	0.1069	1.75	0.04
				Residual	0.7463	0.1388	5.38	<0.0001

Supplemental References

S1. Houde, A. E. *Sex, color, and mate choice in guppies* (Princeton University Press, 1997).

S2. Kuczenski, R. & Segal, D. S. Effects of methylphenidate on extracellular dopamine serotonin, and norepinephrine: comparison with amphetamine. *J. Neurochem*. **68,** 2032-2037 (1997).

S3. Gamo, N. J., Wang, M. & Arnsten, A. F. T. Methylphenidate and atomoxetine enhance prefrontal function through α_2-adrenergic and dopamine D_1 receptors. *J. Am. Acad. Child Adolesc. Psychiatry* **49,** 1011-1023 (2010).

S4. Reznick, D. N. & Endler, J. A. The impact of predation on life history evolution in Trinidadian guppies (*Poecilia reticulata*). *Evolution* **36,** 160-177 (1982).

S5. Rodd, F.H. & Reznick, D. N. Variation in the demography of guppy populations: the importance of predation and life histories. *Ecology* **78,** 405-418 (1997).

S6. Brandstätter, R. & Kotrschal, K. Brain growth patterns in four European cyprinid fish species (Cyprinidae, Teleostei): roach (*Rutilus rutilus*), bream (*Abramis brama*), common carp (*Cyprinus carpio*) and sabre carp (*Pelecus cultratus*). *Brain Behav. Evol*. **35,** 195-211 (1990).

S7. Rodd, F. H. & Sokolowski, M. B. Complex origins of variation in the sexual behaviour of male Trinidadian guppies (*Poecilia reticulata*): interactions among social environment, heredity, body size, and age. *Anim. Behav*. **49,** 1139-1159 (1995).

S8. SAS Institute. *SAS/STAT 9.4 User's Guide* (SAS Institute, 2013).

S9. McDonald, J. H. *Handbook of biological statistics* (Sparky House Publishing, 2014).

S10. Sokal, R. R. & Rohlf, F. J. *Biometry: the principles and practice of statistics in biological research* (W.H. Freeman, 1981).

S11. Husby, A., Saether, B. E., Jensen, H. & Ringsby, T. H. Causes and consequences of adaptive seasonal sex ratio variation in house sparrows. *J. Anim. Ecol.* **75,** 1128-1139 (2006).

S12. R Core Team. *R: A language and environment for statistical computing* (R Foundation for Statistical Computing, 2018).

S13. Burns, J. G., Price, A. C., Thomson, J. D., Hughes, K. A. & Rodd, F. H. Environmental and genetic effects on exploratory behavior of high- and low-predation guppies (*Poecilia reticulata*). *Behav. Ecol. Sociobiol*. **70,** 1187-1196 (2016).

S14. Burns, J. G. The validity of three tests of temperament in guppies (*Poecilia reticulata*). *J. Comp. Psychol*. **122,** 344-356 (2008).

S15. Cachat, J. *et al*. Measuring behavioral and endocrine responses to novelty stress in adult zebrafish. *Nat. Protoc*. **5,** 1786-1799 (2010).

S16. Choleris, E., Thomas, A. W., Kavaliers, M. & Prato, F. S. A detailed ethological analysis of the mouse open field test: effects of diazepam, chlordiazepoxide and an extremely low frequency pulsed magnetic field. *Neurosci. Biobehv. Rev*. **25,** 235-260 (2001).

S17. Levin, E. D., Bencan, Z. & Cerutti, D. T. Anxiolytic effects of nicotine in zebrafish. *Physiol. Behav*. **90,** 54-58 (2007).

S18. Kotrschal, A. *et al*. Artificial selection on relative brain size reveals a positive genetic correlation between brain size and proactive personality in the guppy. *Evolution* **68,** 1139-1149 (2014).

S19. Lê, S., Josse, J. & Husson, F. FactoMineR: An R package for multivariate analysis. *J. Stat. Softw*. **25,** 1-18 (2008).
